# Supplementary material for: Tumour-resident oncolytic bacteria trigger potent anticancer effects through selective intratumoural thrombosis and necrosis
Source: Nat Biomed Eng. 2025 Aug 5;10(2):293–308. doi: 10.1038/s41551-025-01459-9 (PMC12920090; doi:10.1038/s41551-025-01459-9)
Supplement: Supplementary file 1 — Supplementary protocols, references, Figs. 1–46 and Tables 1–9. [file 41551_2025_1459_MOESM1_ESM.pdf]

nature biomedical engineering

Supplementary information https://doi.org/10.1038/s41551-025-01459-9

Tumour-resident oncolytic bacteria trigger potent anticancer effects through selective intratumoural thrombosis and necrosis

In the format provided by the authors and unedited

Contents

Supplementary Protocols

- ***In vitro* anticancer efficacy of bacteria**
- **Transformation of A-gyo**
- **Tumour spheroids**
- **Colony assay**
- **Blood tests**
- **Safety test using lower-limb ischemia model**
- **Intratumour vascular invasion of AUN**
- **Haemolysis test**
- **Coagulation test**
- **Immunohistochemistry staining of tumour and vital organ tissues**
- **Flow cytometry**
- **qPCR**

Supplementary References

Supplementary Figures & Tables

- **Supplementary Figures S1–S46**
- **Supplementary Tables S1–S9**

Supplementary Protocols

***In vitro* anticancer efficacy of bacteria**

To assess *in vitro* anticancer efficacy of UN-gyo, A-gyo, AUN, and homogenized AUN (dead cell), Colon26 cancer cells were pre-seeded for 72 h in 96-well plates at a density of 1×10^4 cells/well. Each concentration of bacteria (5×10^5, 5×10^6, 5×10^7, 5×10^8, and 5×10^9 CFU/mL) was co-cultured with these adhered cells for 24 h in a medium (RPMI 1640:ATCC 543 = 1:1) at 37 °C in a humidified incubator containing 5% CO_2. Thereafter, cells were washed with fresh medium and the viability was measured using a cell counting kit (CCK-8; catalogue no. 346-09271, Dojindo, Kumamoto, Japan). The homogenized AUN (dead cell) was prepared by a pulse-type sonicator (VCX-600; Sonics, Danbury, CT, USA) for 10 min with crushed ice.

Transformation of A-gyo

Colon26 cells were seeded in 96-well plates at a density of 1×10^4 cells/well and allowed to adhere overnight. Cells were then exposed to a medium containing 5×10^7 cells/mL of AUN for 24 h at 37 °C in a humidified incubator containing 5% CO_2. The culture medium consisted of a mixed solution of cell culture medium and bacterial culture medium in a volume ratio of 1:1. After washing thoroughly with fresh PBS solution, the behaviours of bacteria and Colon26 cells were observed using a light microscopy system (BZ-X810; Keyence, Tokyo, Japan) at 20 °C. For the control experiment, AUN alone was cultured in a mixed culture medium of cell and bacteria in a volume ratio of 1:1 for 24 h at 37 °C in a humidified incubator containing 5% CO_2, and cell size and shape were observed by optical microscopy. Oncometabolites including fumaric acid (catalogue no. 16413-52, Nacalai Tesque, Kyoto, Japan), lactic acid (catalogue no. 31604-95, Nacalai Tesque), *O*-phosphorylethanolamine (catalogue no. 27803-31, Nacalai Tesque), and spermidine (catalogue no. 32108-91, Nacalai Tesque), were used to observe the facilitation of A-gyo transformation by optical microscopy. To this end, the AUN was cultured in a mixed culture medium of cell and bacteria in a volume ratio of 1:1 containing each oncometabolite [fumaric acid (0.1 mg/mL), lactic acid (1 mg/mL), *O*-phosphorylethanolamine (1 mg/mL), and spermidine (1 mg/mL)] for 24 h at 37 °C.

For observation of A-gyo swarmers and thrombosis in tumour tissues, the Colon26 tumour–bearing BALB/c-nu/nu mice (female; about 7 weeks old; average weight = 20 g; average tumour size = 200 mm^3; n = 3; genetic background: BALB/c; substrain: BALB/cCrSlc-nu/nu; obtained from Japan SLC) were euthanized 24 h after the administration of PBS (200 µL) or AUN (200 µL, 7.8×10^9 CFU/mL) i.v. injection. Tumours and blood were collected from the AUN- or PBS-treated mice and the normal mouse without any treatments, respectively. The sliced tumour tissues and coagulated blood samples were observed using a light microscopy system (BZ-X810) at 20 °C.

Tumour spheroids

Colon26 cells (1×10^4 cells/well) were seeded in a 3D culture spheroid plate (Cell-able® BP-96-R800; Toyo Gosei, Tokyo, Japan) according to the manufacturer's instructions provided with the plate. Cells were cultured for 5 days at 37 °C in a humidified incubator containing 5% CO_2. The medium was replaced every 2 days. Prepared spheroids were then exposed to a mixed culture medium (cell culture medium:bacterial culture medium = 1:1) containing 1×10^7 CFU of AUN for 0, 24, 48, 72, 96, and 120 h at 37 °C in a humidified incubator containing 5% CO_2. After washing thoroughly with fresh PBS solution, spheroids were observed using the BZ-X810 at 20 °C. A control experiment without AUN was performed also.

Colony assay

The BALB/c-nu/nu mice (female, 6 weeks old; average weight = 18 g; n = 3; genetic background: BALB/c; substrain:

BALB/cCrSlc-nu/nu; obtained from Japan SLC) were i.v. injected in the tail vein with culture medium (200 μL) containing AUN (1×10^7 CFU/mL or 1×10^9 CFU/mL). Mice given the lower concentration of AUN were given a second dose of AUN (200 μL, 1×10^9 CFU/mL) by i.v. 2 days later. Control experiments were also performed using PBS (200 μL). Blood samples were collected from the inferior vena cava of the mice after 5 min and 6 h. Each blood sample (100 μL) was inoculated onto an agar plate. After being anaerobically incubated for 7 days, the bacterial colonies that formed were imaged. To count the bacterial colonies, the supernatant was diluted 0, 10, 100, and 1000 times with PBS, and then a sample (5 μL) was inoculated onto an agar plate as mentioned above. Finally, bacterial colonies that formed were manually counted.

Blood tests

The complete blood count was measured using the Celltac α automated clinical haematology machine (MEK6558; Nihon Kohden, Tokyo, Japan), and biochemical parameters were investigated by Oriental Yeast Co. (Tokyo, Japan). The cytokines were measured by Bio-Plex Multiplex immunoassay system (Bio-Rad, Hercules, CA, USA) and Bio-Plex Pro Mouse Th17 Panel 6-Plex (Bio-Rad). The factor VII activity was measured using Facteur VII Chromogenic Assay BIOPHEN FVII kit (catalogue no. A221304, Hyphen-BioMed, Neuville sur Oise, France). BALB/c-nu/nu mice (female; 6 weeks old; average weight = 18 g; n = 5; genetic background: BALB/c; substrain: BALB/cCrSlc-nu/nu; obtained from Japan SLC, Shizuoka, Japan) were injected in the tail vein with culture medium containing AUN (200 μL, 7.8×10^9 CFU/mL or 1.0×10^7 CFU/mL) or PBS (200 μL). Mice given the lower dose of AUN were then given a second dose of AUN (200 μL, 15×10^9 CFU/mL) 48 h later. The blood samples were collected from the inferior vena cava of the mouse at 3, 6, 24, 48, 72, and 240 h after administration of the second dose. Control experiments without administration of bacteria were also performed as 0 h treatment.

The concentration of immunoglobulins (IgG and IgM) in blood plasma was measured by Oriental Yeast Co. using Mouse IgG ELISA kit (catalogue no. E99-131, Bethyl Laboratories, Montgomery, TX, USA) and Mouse IgM ELISA kit (catalogue no. E99-101, Bethyl Laboratories). For the test, the blood samples were collected from the inferior vena cava of BALB/cCrSlc-nu/nu mice (female; 6 weeks; n = 5; average weight = 18 g; Japan SLC) after i.v. injection of AUN (200 μL, 1.0×10^7 CFU/mL) for 48 h. Blood plasma was then obtained by centrifugation at $1110 \times g$ for 5 min at 4 °C (MX-310; Tomy, Tokyo, Japan). Control blood samples were also obtained from non-treated BALB/cCrSlc-nu/nu mice (female; 6 weeks old; average weight = 18 g; n = 5; genetic background: BALB/c; substrain: BALB/cCrSlc-nu/nu; obtained from Japan SLC).

Safety test using lower-limb ischemia model

Lower-limb ischemia model mice were prepared by Japan SLC. Briefly, the right femoral artery and vein of BALB/c mice (female; 6 weeks old; average weight = 18 g; n = 5; substrain: BALB/cCrSlc; genetic background: BALB/c; obtained from Japan SLC) were exposed and resected from the proximal portion near the inguinal ligament to the distal portion of the saphenous artery. The remaining arterial branches were also excised. The contralateral hind limb served as an internal control. All surgeries were performed under general anaesthesia. The model mice (8 weeks old; average weight = 20 g; n = 3) were i.v. injected in the tail vein with culture medium (200 μL) containing AUN (5×10^9 CFU/mL) or PBS (200 μL). Blood and vital organs were collected from the model mice 30 days after i.v. administration of samples for further blood test and histological examination. Body weights of the mice were also measured for 10 days.

Intratumour vascular invasion of AUN

To monitor the intratumour vascular invasion of AUN, IR-1061-modified AUN and NIR-II FL bio-imager were used. IR-

1061-modified AUN was prepared by referring to the previous methods.[1,2] Briefly, 0.1 mg/mL of IR-1061 (catalogue no. 405124, Sigma-Aldrich, St. Louis, MO, USA) was dissolved in saline with 10% Cremophor® EL and 10% DMSO (catalogue no. 043-07216, FUJIFILM Wako Pure Chemical, Osaka, Japan) using sonication to obtain IR-1061 dispersion. The medium containing AUN was centrifuged at 820 × *g* for 5 min at a temperature of 4 °C and washed with PBS. Following this, the concentration of the bacterial suspension was adjusted to 2 × 10^9 CFU/mL and centrifuged to obtain a bacterial pellet. IR-1061 dispersion (IR-1061 concentration = 0.1 mg/mL) was added, and the bacterial suspension was incubated 1 h at 37 °C. Further, the samples were centrifuged to remove the unattached dye molecules, and the modified bacteria were resuspended in saline. Colon26 tumour–bearing BALB/c-nu/nu mice (female; about 7 weeks old; average weight = 20 g; average tumour size = 200 mm^3; n = 3; genetic background: BALB/c; substrain: BALB/cCrSlc-nu/nu; obtained from Japan SLC) were injected intravenously with saline containing IR-1061-modified AUN (200 µL, 2 × 10^9 CFU/mL) or PBS (200 µL). NIR-II fluorescence of the treated mice were observed using NIR-II fluorescent bio-imager (SAI-1000; Shimazu, Kyoto, Japan).

Haemolysis test

Human blood sample including anticoagulants (catalogue no. CTSAG030020; Human Heparin sodium whole blood, single donor) was purchased from BioIVT (College Park, MD, USA). Haemolysis test was surveyed by referencing the previous methods.[3,4] Briefly, the blood (100 µL) was diluted 5 times by saline. The diluted blood (500 µL) was then mixed with different concentrations of bacterial dispersion (50 µL, 5 × 10^3 CFU/mL, 5 × 10^4 CFU/mL, 5 × 10^5 CFU/mL, 5 × 10^6 CFU/mL, 5 × 10^7 CFU/mL, and 5 × 10^8 CFU/mL), saline (negative control) (50 µL), or RIPA lysis buffer (positive control) (catalogue no. 16488-34, Nacalai Tesque) (50 µL). After incubation for 6 h at 37°C, the samples were centrifuged at 820 × *g* for 5 min at 4 °C. The supernatants were carefully collected, and their absorbance at 576 nm (A) were measured by a microplate reader. Finally, the values obtained from samples treated with bacteria (A_{bac}) were normalized relative to positive (100% lysis; A_{pos}) and negative (saline; A_{neg}) control samples to provide the haemolysis ratio (%) by using the following equation:

$$\text{Haemolysis ratio (\%)} = (A_{bac} - A_{neg})/(A_{pos} - A_{neg}) \times 100$$

Coagulation test

To evaluate whether AUN, A-gyo, UN-gyo, and homogenized AUN have thrombus formation ability, coagulase test was performed with rabbit plasma kit (catalogue no. E-ME07, Eiken Chemical, Tokyo, Japan) according to the manufacturer's instructions provided with the kit and referencing the previous methods.[5] *Staphylococcus aureus* was used as a positive control. Briefly, for the slide test, a droplet of saline (ca. 10 µL) was casted on a microscope slide, and centrifuged bacterial pellet, homogenized AUN dispersion (ca. 10 µL, bacterial concentration is about 1 × 10^{10} CFU/mL), or saline (ca. 10 µL) was then mixed with the saline on a slide. A droplet of plasma solution (ca. 10 µL) was mixed with each solution, and carefully observed them for 5 min to confirm coagulation. Besides, for the tube test, rabbit plasma (1 mL) was mixed with bacterial suspension (50 µL, 5 × 10^9 CFU/mL), homogenized AUN dispersion (50 µL, 5 × 10^9 CFU/mL), or saline (50 µL) in a test tube, and incubated at 37°C for 24 h to carefully observe coagulation.

Immunohistochemistry staining of tumour and vital organ tissues

The Colon26 tumour–bearing BALB/c-nu/nu mice (female; about 8 weeks old; average weight = 20 g; average tumour size = 400 mm^3; n = 3; genetic background: BALB/c; substrain: BALB/cCrSlc-nu/nu; obtained from Japan SLC) were euthanized the day after or at 10 days after the administration of PBS (200 µL) or AUN (200 µL, 7.8 × 10^9 CFU/mL) i.v. injection. The orthotopic pancreatic tumour–bearing BxPC3 mice (male; 11 weeks old; average weight = 34 g; n = 5; genetic background:

BALB/c; substrain: BALB/cAJcl-nu/nu; obtained from CLEA Japan, Tokyo, Japan) were also euthanized 10 days after a single dose of PBS (200 μL) or of two doses of AUN (1st dose: 200 μL, 1.0 × 10^9 CFU/mL, 2nd dose: 200 μL, 20 × 10^9 CFU/mL) given i.v. Then, the tumour and vital organ tissues from the different treatment groups were harvested for IHC staining. Analysis was performed by Biopathology Institute Co., Ltd. (Oita, Japan) using standard protocols. Briefly, primary tumours were surgically removed, fixed in 10% formalin, processed for paraffin embedding, and cut into 3–4-μm-thick sections. After incubation with primary antibodies (**listed in Table S7, Supplementary Information**), the sections were stained with haematoxylin and examined using light microscopy (IX73). The areas showing positive staining in tumour tissues were analysed using a light microscopy system (BZ-X810) and a hybrid cell count and microcell count software (BZ-X800 Analyser V1.1.2.4; Keyence).

Specimen preparations and analyses for bacterial location in tumours of AUN-treated mice after administration with and without heparin were performed by Daiichi Sankyo RD Novare Co., Ltd. (Tokyo, Japan). Briefly, AUN (200 μL, 5 × 10^9 CFU/mL) was i.v. injected to Colon26-bearing BALB/c-nu/nu mice (female; about 7 weeks old; average weight = 20 g; average tumour size ~200 mm^3; n = 3; genetic background: BALB/c; substrain: BALB/cCrSlc-nu/nu; obtained from Japan SLC) with or without administration of heparin (5 mg/head) for 1 h. Tumour tissues were collected from different groups of mice at 24 h after treatments for haematoxylin and eosin staining.

Flow cytometry

The Colon26 tumour–bearing mice (female; about 8 weeks old; average weight = 18 g; average tumour size = 400 mm^3; n = 3; genetic background: BALB/c; substrain: BALB/cCrSlc-nu/nu; obtained from Japan SLC) were euthanized the day after i.v. injection of PBS (200 μL) or AUN (200 μL, 7.8 × 10^9 CFU/mL). To analyse the immune cells in tumours, tumours were collected from mice in different groups at 24 h after i.v. injection of sample and homogenized into single-cell suspensions (gentleMACS Octo Dissociator with Heaters; Miltenyi Biotec , Bergisch Gladbach, Germany). Then, red blood cells were removed by Red Blood Cell Lysis Solution (catalogue no. 130-094-183, Miltenyi Biotec), and debris were removed by cell strainer (mesh size = 40 μm, SureStrain; MTC Bio, Sayreville, NJ, USA). Samples containing 1 × 10^6 cells were stained with antibodies labelled with FITC, PE, PerCP-Vio 700, PE-Vio 770, APC, or APC-Vio 770 (**listed in Table S7, Supplementary Information**) according to the manufacturer's protocols, and then classified by flow cytometry (MACSQuant® Analyser 16; Miltenyi Biotec, Bergisch Gladbach, Germany) and software (MACSQuantify V2.13.3; Miltenyi Biotec). Spleens were also analysed 2 days after an i.v. injection of a low dose of AUN (1 × 10^7 CFU/mL).

qPCR

The Colon26 tumour–bearing mice (female; about 8 weeks old; average weight = 18 g; average tumour size = 400 mm^3; n = 3; genetic background: BALB/c; substrain: BALB/cCrSlc-nu/nu; obtained from Japan SLC) were euthanized the day after i.v. injection of PBS (200 μL) or AUN (200 μL, 7.8 × 10^9 CFU/mL). To analyse the immune cells and cytokines in tumours, tumours were collected from mice in different groups 24 h after i.v. injection of sample and then homogenized by a handy homogenizer (Thermo Fisher Scientific) before qPCR. qPCR was performed using QuantStudio 1 PCR system (Thermo Fisher Scientific) to study the relative gene expression of CD3, CD19, CXCR4, F4/80, NK, IFN-γ, and TNF-α, using gene-specific primer–probe combinations (Thermo Fisher Scientific) (**listed in Table S8, Supplementary Information**) by Taqman chemistry. Endogenous control was determined using TaqMan Array Mouse Endogenous Control Plate, 96-well (Thermo Fisher Scientific). The reactions were run in triplicate using GAPDH as an endogenous control. The thermal cycling parameters that yielded optimum amplification were 50 °C for 2 min for AmpErase UNG activation, 95 °C for 2 min for

AmpliTaq Gold DNA Pol. activation and 40 cycles each of 95 °C for 1 s for denaturation/melting and 60 °C for 20 s for annealing and extension. For the test genes and endogenous control standards, 10-fold serial dilutions were run in the study to estimate the efficiency of PCR and the percentage efficiency ranged between 90–100%. Results were analysed and shown as fold change (log_{10} relative quantification) relative to the control group.

Bacterial genomic DNA was extracted from frozen mouse tumours and cultured bacteria using the NucleoSpin® Microbial DNA kit (catalogue no. U0235B, TaKaRa, Kyoto, Japan) according to the manufacturer's protocol. The 16S rDNA primers (Eurofins Genomics, Tokyo, Japan) used for RT-qPCR are listed in **Table S9, Supplementary Information**. RT-qPCR was performed using TB Green Premix Ex Taq II (Tli RNase H Plus; catalogue no. RR820A, TaKaRa, Kyoto, Japan) and a QuantStudio 1 PCR System. Amplification data were normalized to the amplification levels of each primer.

Supplementary References

1. Goto, Y., Iwata, S., Miyahara, M. & Miyako, E. Discovery of intratumoral oncolytic bacteria toward targeted anticancer theranostics. *Adv. Sci.* **10**, 23016 (2023).
2. Reghu, S. & Miyako, E. Nanoengineered *Bifidobacterium bifidum* with optical activity for photothermal cancer immunotheranostics. *Nano Lett.* **22**, 1880–1888 (2022).
3. Sæbø, I. P., Bjørås, M., Franzyk, H., Helgesen, E. & Booth, J. A. Optimization of the hemolysis assay for the assessment of cytotoxicity. *Int. J. Mol. Sci.* **24**, 2914 (2023).
4. Mesdaghinia, A., Pourpak, Z., Naddafi, K., Nodehi, R. N., Alizadeh, Z., Rezaei, S., Mohammadi, A. & Faraji, M. An *in vitro* method to evaluate hemolysis of human red blood cells (RBCs) treated by airborne particulate matter (PM10). *MethodsX* **6**, 156–161 (2019).
5. Katz, D. S. Coagulase test protocol. American Society for Microbiology Laboratory Protocols (2010) https://asm.org/Protocols/Coagulase-Test-Protocol

Supplementary Figures & Tables

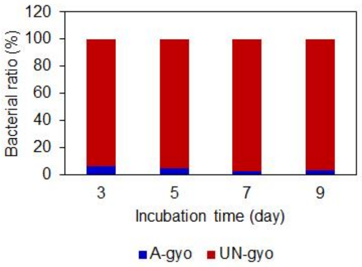

Supplementary Figure S1. Bacterial ratios of A-gyo and UN-gyo in AUN during culturing at the different time points (Day 3, Day 5, Day 7, and Day 9). The repeated subcultured and cryopreserved AUN was used for the test. Data are represented as average values; n = 3 independent experiments.

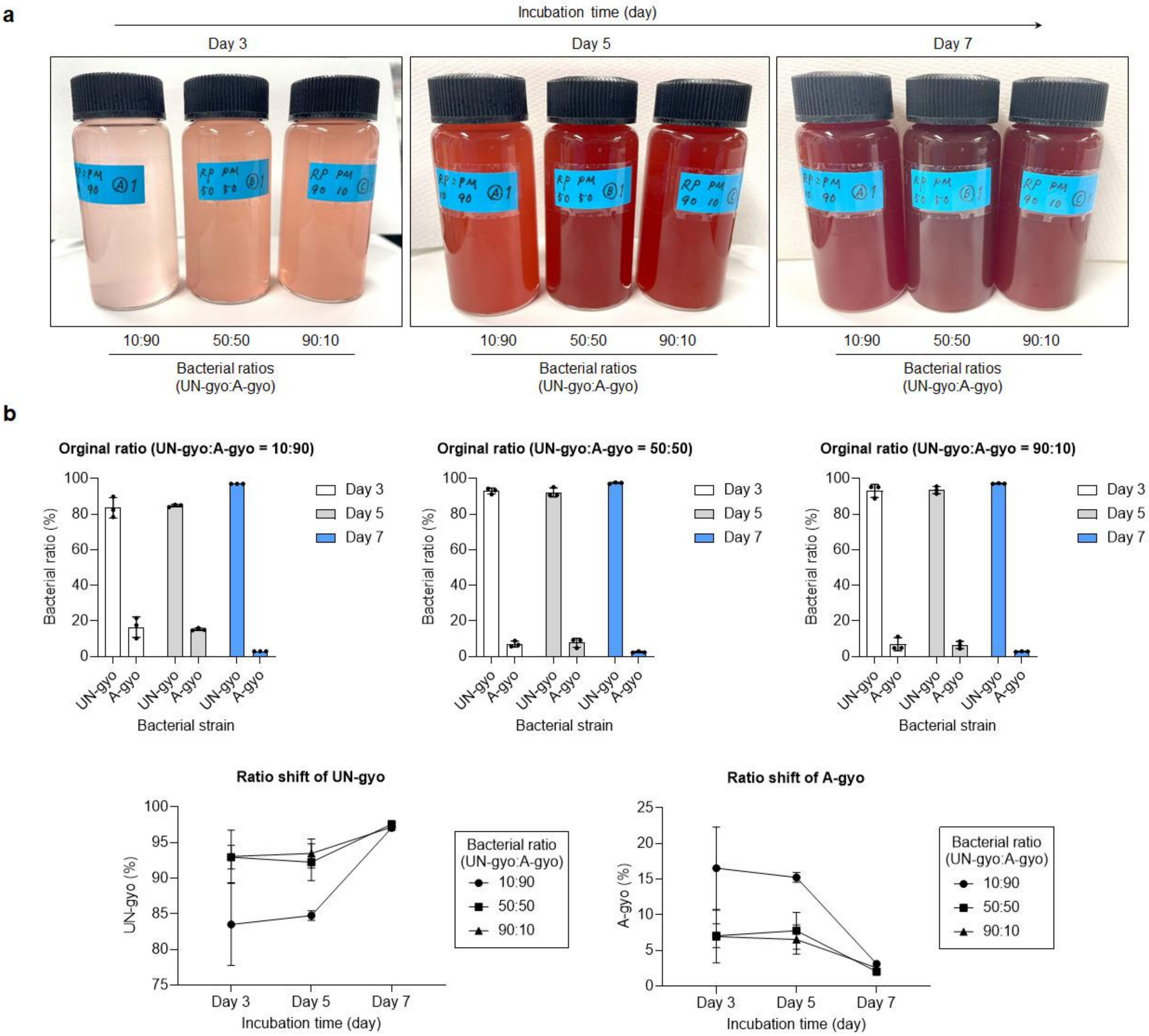

Supplementary Figure S2. Bacterial ratio shifts of the artificial mixed AUN suspensions after culturing for 7 days. (a) Photos of cultured bacterial suspensions at each time points (Day 3, Day 5, and Day 7). (b) Bacterial ratio shifts of the AUN after culturing for 7 days. The mixed ratio was adjusted to UN-gyo:A-gyo = 10:90, 50:50, or 90:10 before culturing. Data are represented as mean ± SEM; n = 3 independent experiments.

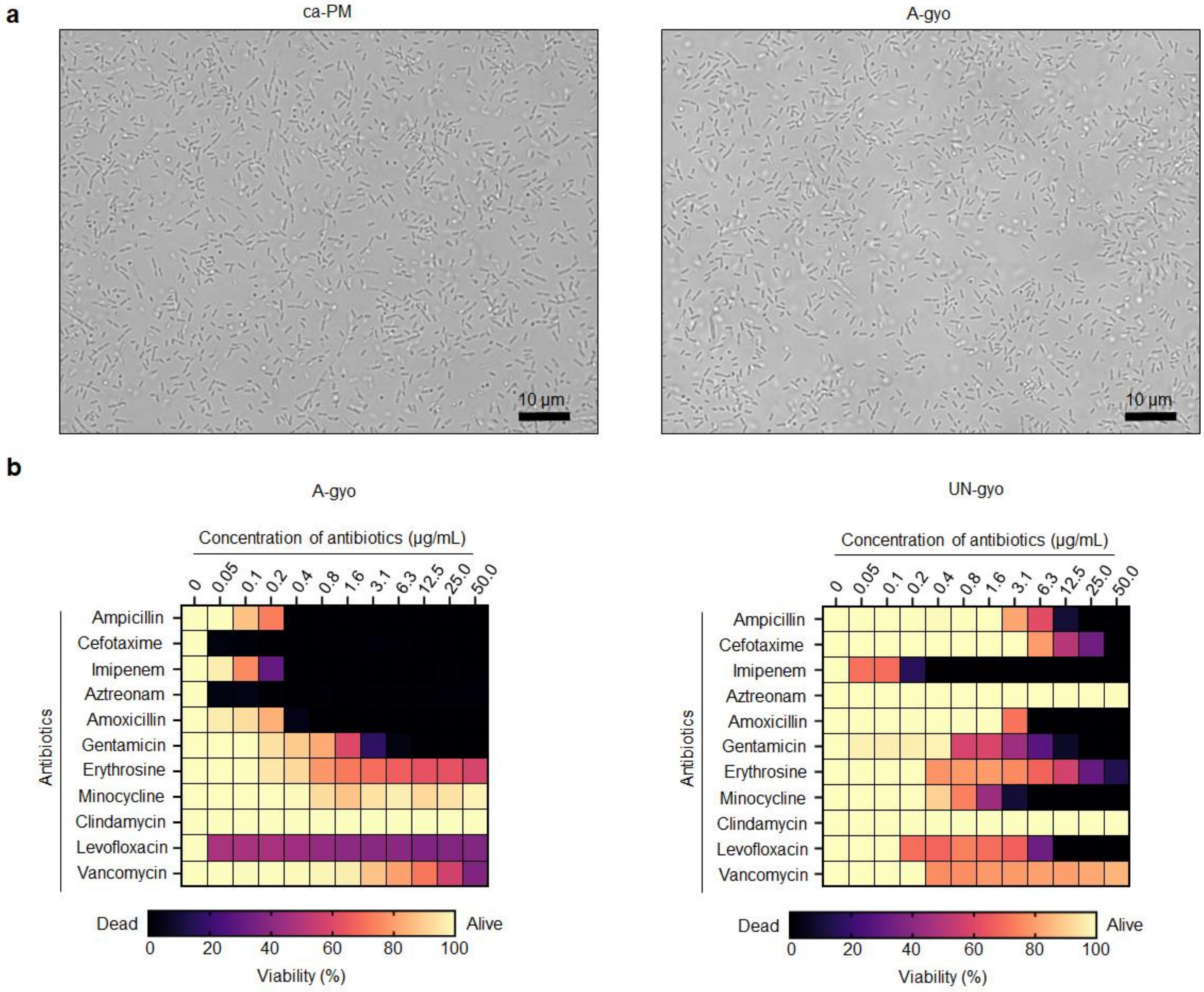

Supplementary Figure S3. Characterizations of A-gyo and UN-gyo. (a) Differential interference contrast imaging of *Proteus mirabilis* (ca-PM) (left) and A-gyo (right). n = 3 independent experiments. (b) Minimum inhibitory concentration test of various antibiotics against A-gyo (left) and UN-gyo (right). Data are represented as mean ± SEM; n = 3 independent experiments.

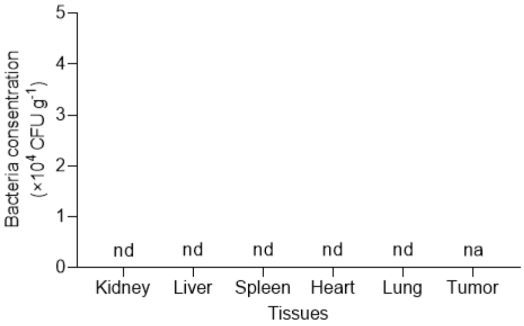

Supplementary Figure S4. Numbers of the bacterial colony of organs and tumour in Colon26 tumour–bearing BALB/c-nu/nu mice after administration of AUN and antibiotics imipenem. Saline (200 µL) containing imipenem (1 mg/head) was i.p. injected into BALB/c-nu/nu mice 24 h after i.v. administration of AUN (200 µL, 5×10^9 CFU/mL). After 24 h, the organs and tumours were carefully excised and weighed for further colony assay. n = 5 independent experiments. nd, not detectable. na, not available due to the complete disappearances of tumours 24 h after AUN injection.

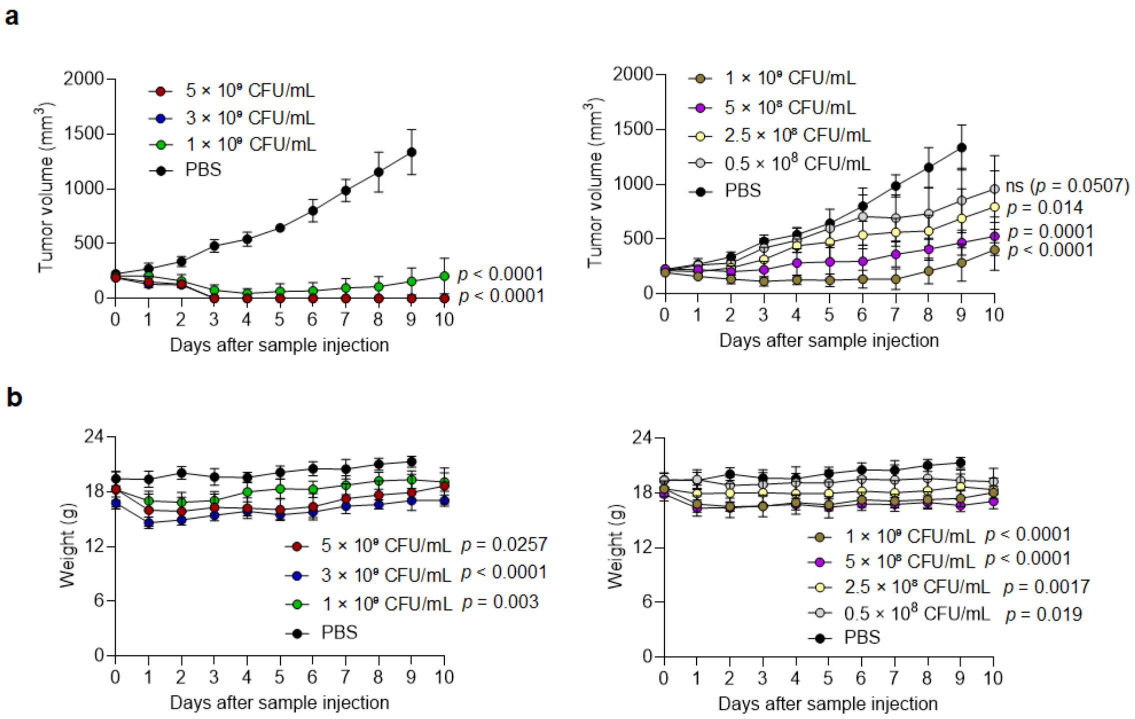

Supplementary Figure S5. *In vivo* anticancer effect of AUN at different concentrations. (a) The suspension of AUN was i.v. injected once into Colon26-bearing BALB/c-nu/nu mice. A control experiment (a single i.v. dose of PBS) was performed also. Data are represented as mean ± standard error of the mean (SEM); n = 5 biologically independent mice. Statistical significance of the single-dose group at the endpoint was calculated by comparison with PBS group. ns, not significant; *p*-values were obtained using Student's two-sided *t*-test. (b) Body weight measured daily after each treatment (single dose of AUN and single dose of PBS) in Colon26 tumour–bearing BALB/c-nu/nu mice. Data are represented as mean ± SEM; n = 5 biologically independent mice. Statistical significance of the single-dose group at the endpoint was calculated by comparison with the PBS group. *p*-values were obtained using Student's two-sided *t*-test.

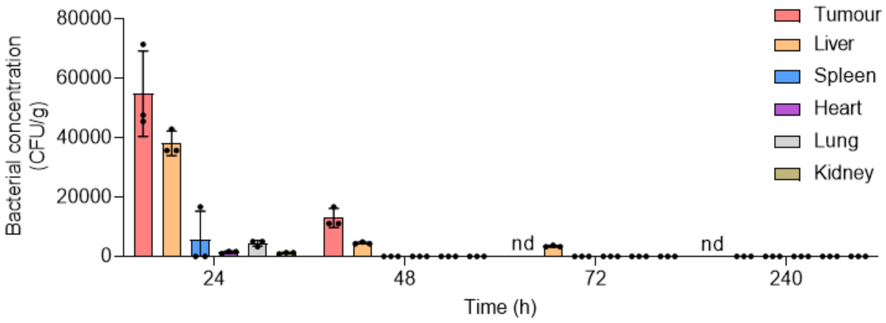

Supplementary Figure S6. Biocompatibility of AUN. Number of bacterial colonies in organs of Colon26 tumour–bearing BALB/c-nu/nu mice 24, 48, 72, and 240 h after a single shot of AUN (7.8×10^9 CFU/mL). Data are represented as mean ± standard error of the mean; n = 3 independent experiments. nd, not detectable because tumours had totally disappeared.

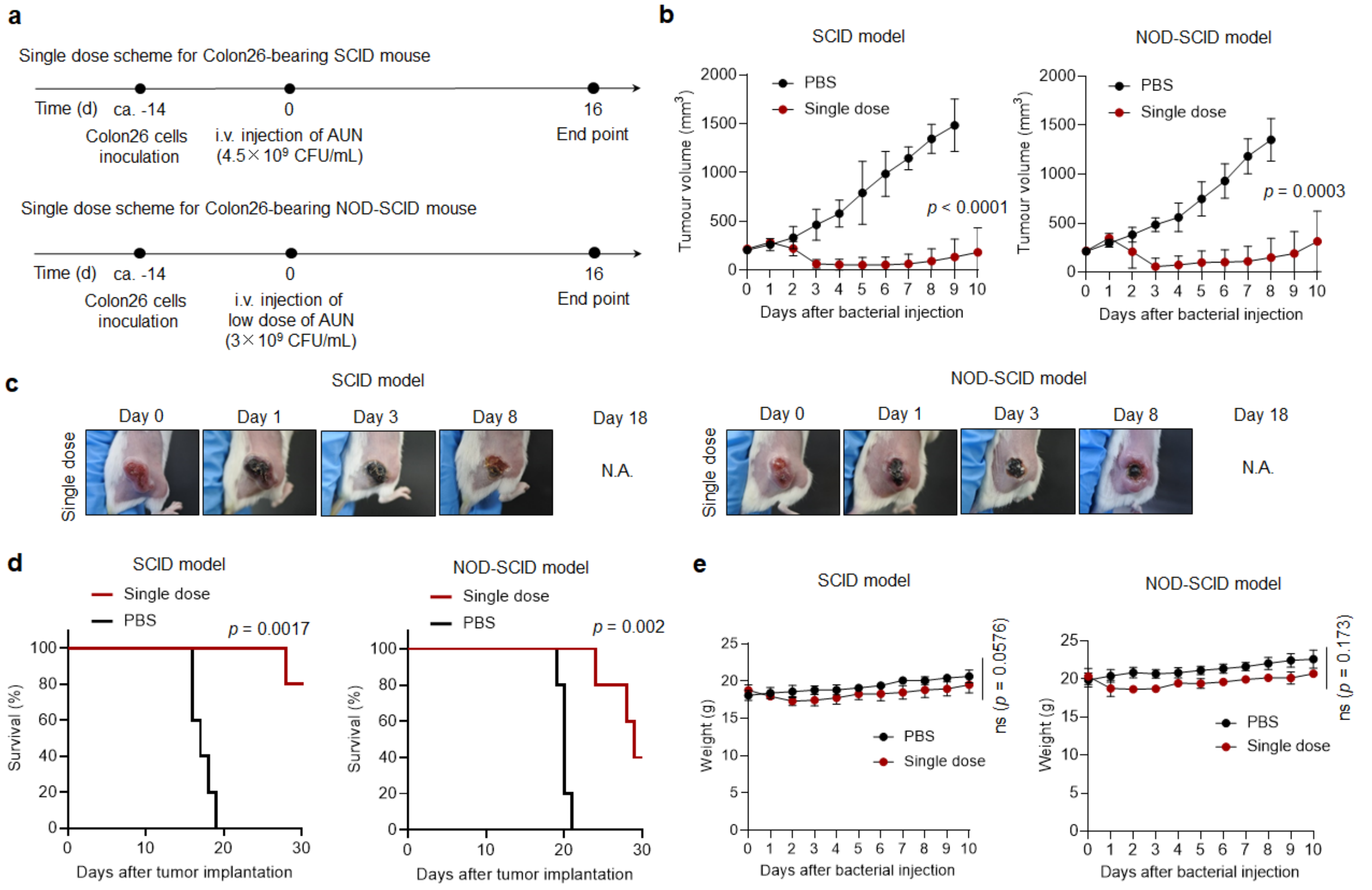

Supplementary Figure S7. Antitumour efficacy of a single dose of AUN to severe combined immunodeficient (SCID) and non-obese diabetic (NOD)-SCID mice bearing Colon26 tumours. (a) Time course of establishment and treatment of Colon26 tumour–bearing SCID and NOD-SCID mice. After tumour establishment, mice were intravenously injected with a single dose of AUN. (b) *In vivo* anticancer effect of a single dose of AUN (4.5×10^9 CFU/mL for SCID and 3.0×10^9 CFU/mL for NOD-SCID) by i.v. injection. The control experiment (a single intravenous dose of PBS) was also performed. Data are represented as mean ± SEM; n = 5 biologically independent mice. Statistical significance at the endpoint was calculated by comparison with the PBS group. *p*-values were obtained using Student's two-sided *t*-test. (c) Images of mice after each treatment. N. A., not available. (d) Kaplan–Meier survival curves of Colon26 tumour–bearing SCID and NOD-SCID mice (n = 5 biologically independent mice) for 30 days after tumour implantation. Statistical significance at the endpoint was calculated by comparison with the PBS group. *p* values were obtained using Log-rank (Mantel-Cox) test. (e) Body weight measured daily after the single dose in Colon26 tumour–bearing SCID and NOD-SCID mice. Data are represented as mean ± standard error of the mean; n = 5 biologically independent mice. Statistical significance was calculated by comparison with the PBS group. ns, not significant; *p*-values were obtained using two-way ANOVA test.

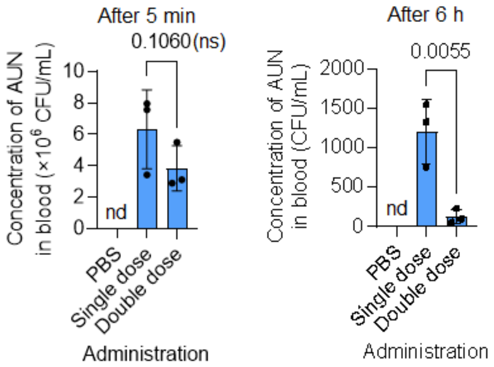

Supplementary Figure S8. Colony assay in blood samples of BALB/c-nu/nu mice taken 5 min (left) and 6 h (right) after treatment with AUN. The concentration of administered AUN was 1×10^9 CFU/mL. Data are represented as mean ± standard error of the mean; n = 3 biologically independent mice. nd, not detectable; ns, not significant; *p*-values were obtained using Student's two-sided *t*-test.

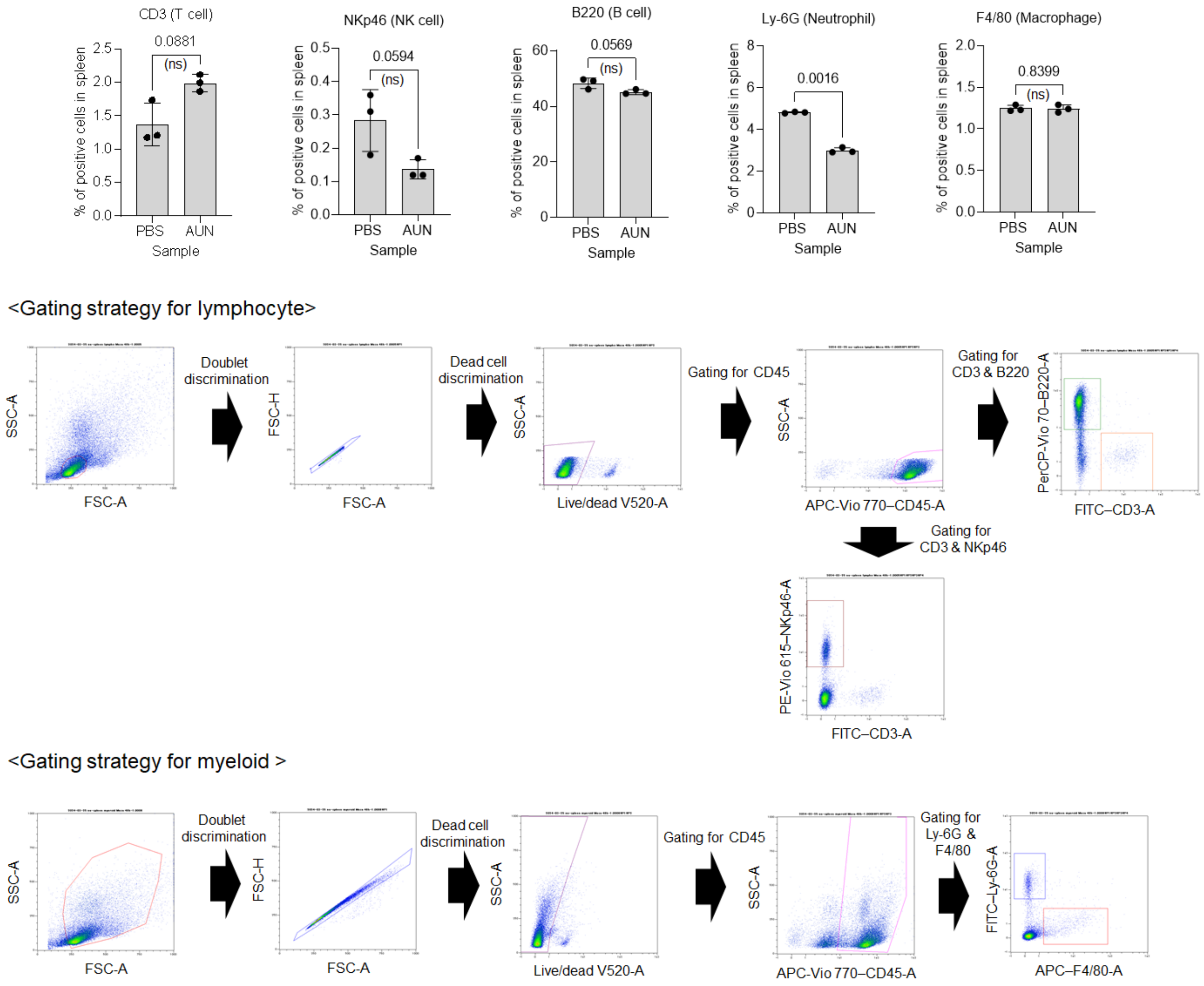

Supplementary Figure S9. Flow cytometry analyses of expression of CD3+, NKp46+, B220+, Ly-6G+, and F4/80 cells in spleen of BALB/c-nu/nu mice after intravenous injection of each sample for 48 h. The concentration of administered AUN was 1.0×10^7 CFU/mL. Data are represented as mean ± standard error of the mean; n = 3 independent experiments. Statistical significance was calculated in comparison with the non-treatment group. ns, not significant; *p*-values were obtained using Student's two-sided *t*-test. Gating strategy indicates the representative example for identification of lymphocyte and myeloid cells.

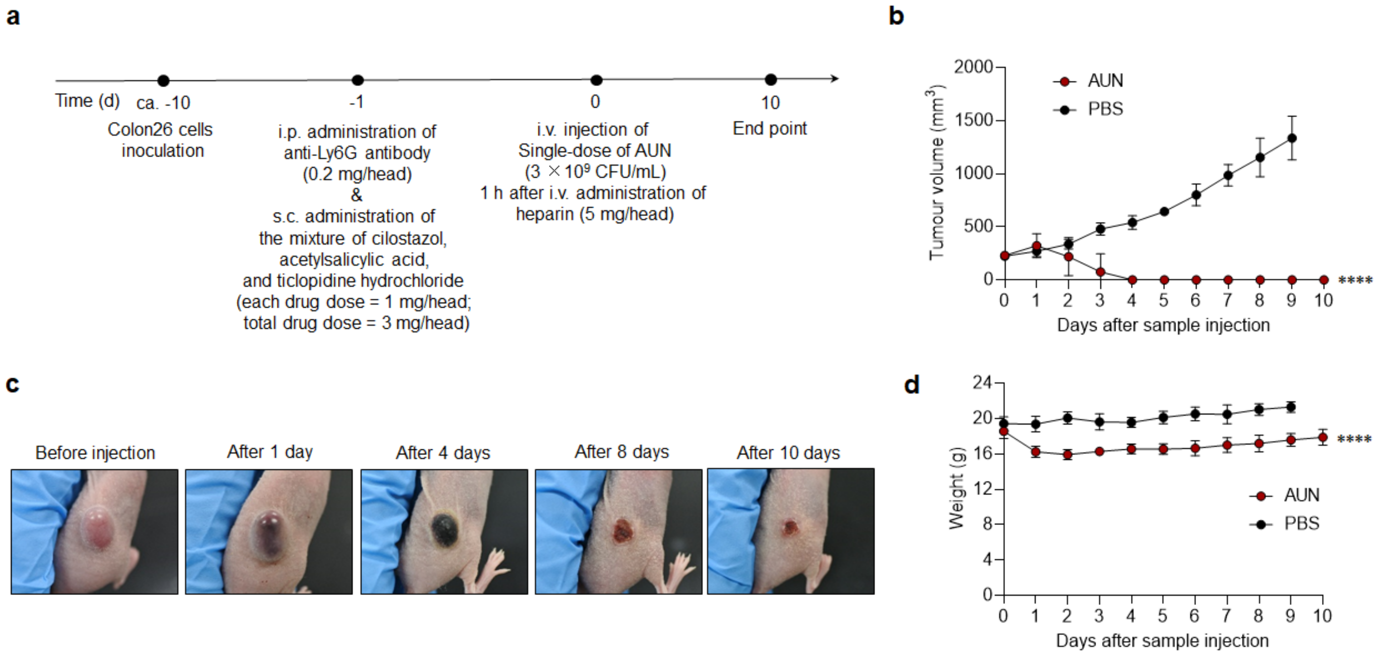

Supplementary Figure S10. Antitumour efficacy of AUN in Colon26 tumour–bearing BALB/c-nu/nu mice previously treated with anti-Ly6G antibody and the combination of antiplatelet drugs and heparin. (a) Time course of establishment and treatment of mice. After tumour establishment, mice were intravenously (i.v.) injected with heparin and AUN in accordance with the time course. (b) *In vivo* anticancer effect of AUN in Colon26 tumour–bearing BALB/c-nu/nu mice after administration with anti-Ly6G antibody (0.2 mg/head) and the mixture of three antiplatelet drugs (each drug dose = 1 mg/head; total dose = 3 mg/head) and heparin (5 mg/head). The suspension of AUN (3.0×10^9 CFU/mL) was i.v. injected once. Statistical significance at the endpoint was calculated by comparison with the PBS group. Data are represented as the mean ± standard errors of the mean (SEM); n = 5 biologically independent mice. ****, $p < 0.0001$, by Student's two-sided *t*-test. (c) Images of mice after the treatment of AUN. (d) Body weight of mice was measured daily after the treatment followed by a single dose of AUN (3.0×10^9 CFU/mL). Statistical significance at the endpoint was calculated by comparison with the PBS group. Data are represented as the mean ± SEM; n = 5 biologically independent mice. ****, $p < 0.0001$, by Student's two-sided *t*-test.

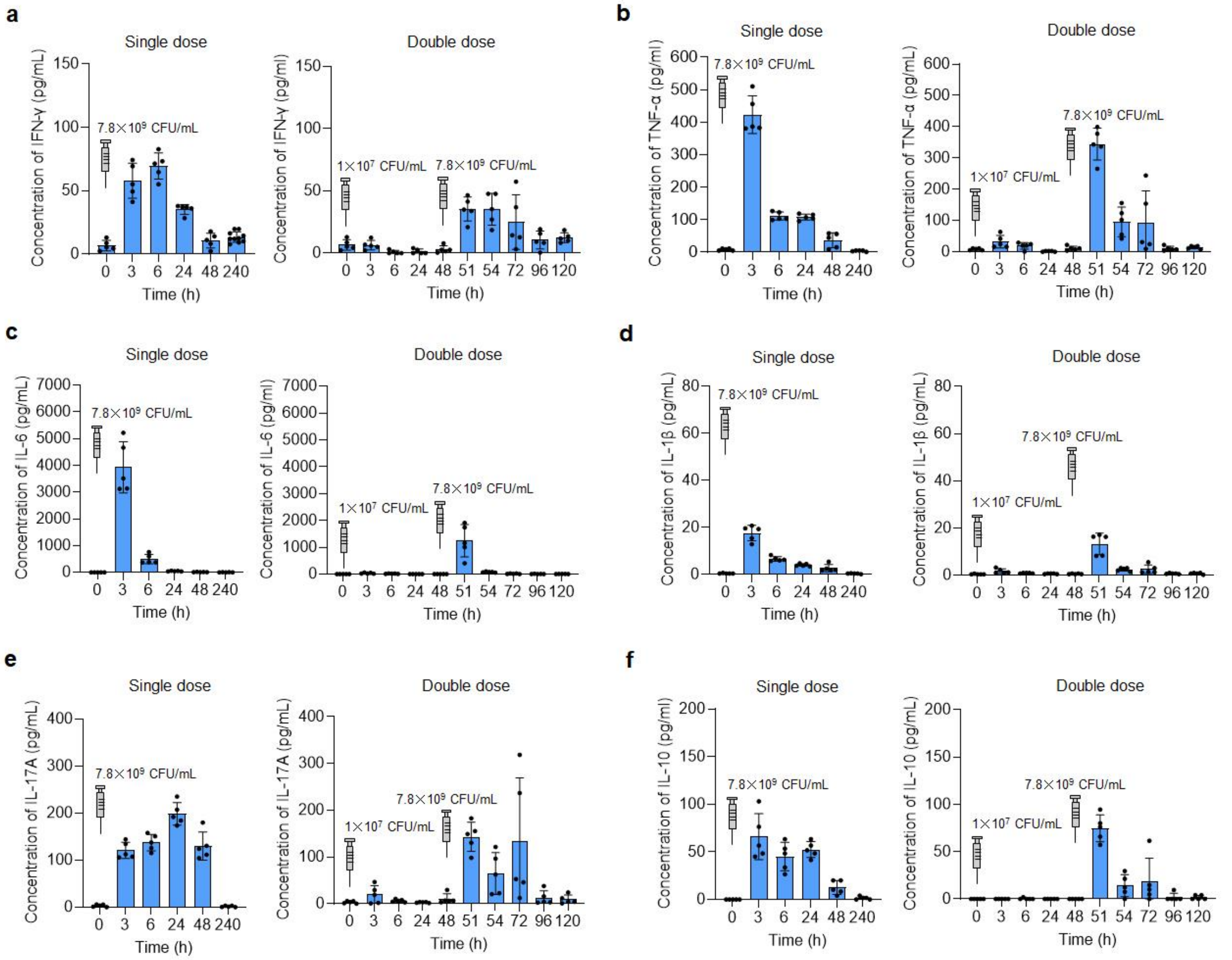

Supplementary Figure S11. Cytokine assays in blood of BALB/c-nu/nu mice after a single dose (left) or a double dose of AUN (right) for measurement of (a) IFN-γ, (b) TNF-α, (c) IL-6, (d) IL-1β, (e) IL-17A, and (f) IL-10. The concentration of AUN in the single dose was 7.8 × 10^9 CFU/mL. For two doses, the AUN concentrations of the 1st and 2nd doses were 1 × 10^7 CFU/mL and 7.8 × 10^9 CFU/mL, respectively. Data are represented as mean ± standard error of the mean; n = 5 biologically independent mice.

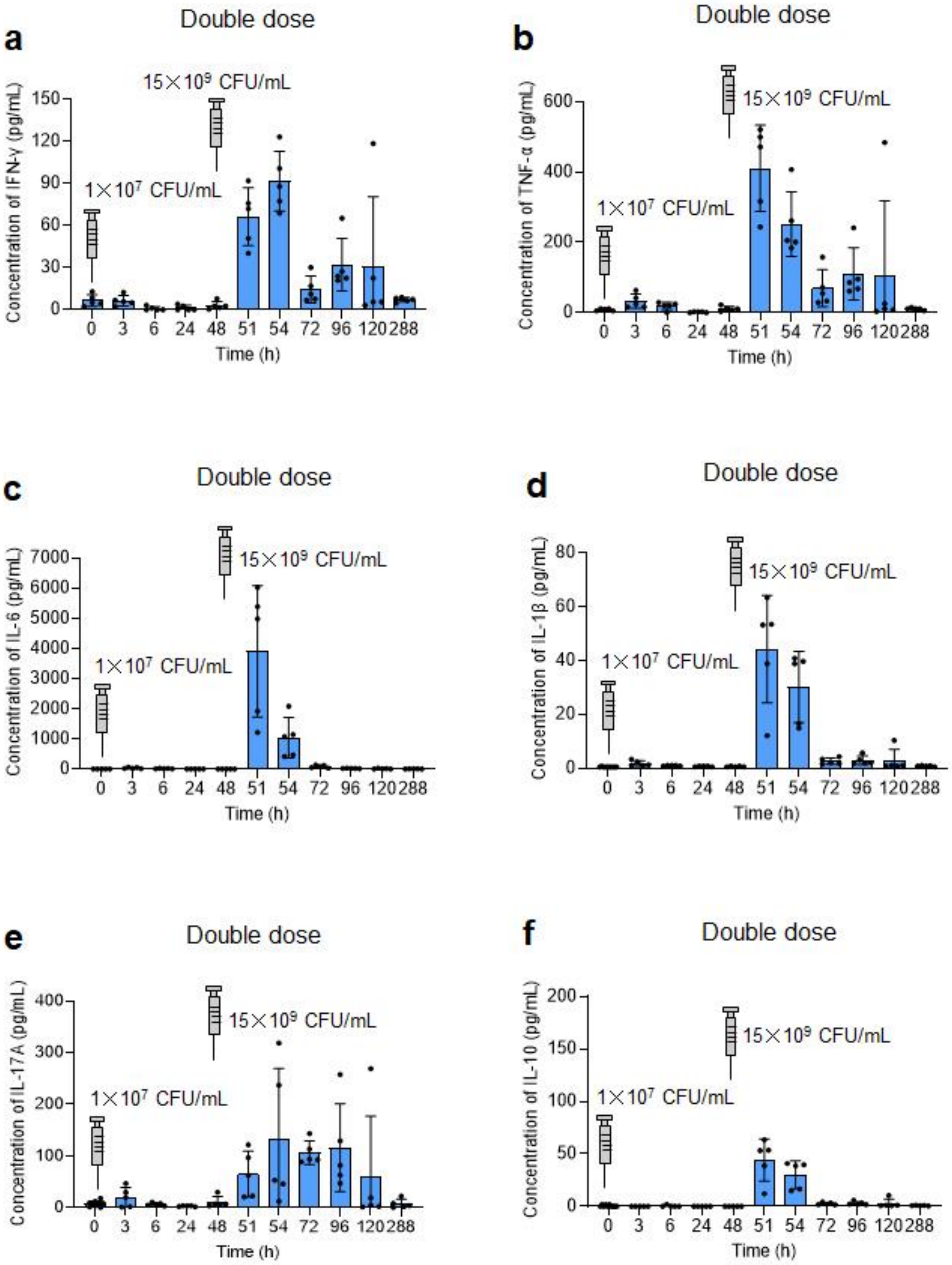

Supplementary Figure S12. Cytokine assays in blood of BALB/c-nu/nu mice after two doses of AUN for measurement of (a) IFN-γ, (b) TNF-α, (c) IL-6, (d) IL-1β, (e) IL-17A, and (f) IL-10. Data are represented as mean ± standard error of the mean; n = 5 biologically independent mice.

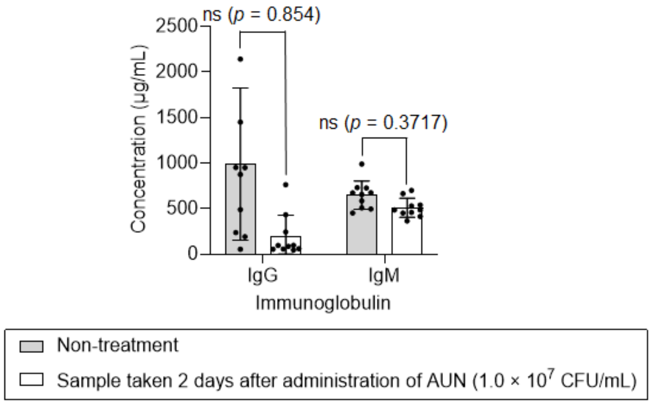

Supplementary Figure S13. Concentration of bacterially sensitive immunoglobulins (IgG and IgM) in blood of BALB/c-nu/nu mice 2 days after intravenous administration of AUN. The concentration of administered AUN was 1.0×10^7 CFU/mL. ns, not significant; *p*-values were obtained using Student's two-sided *t*-test. Data are represented as mean ± standard error of the mean; n = 10 biologically independent mice.

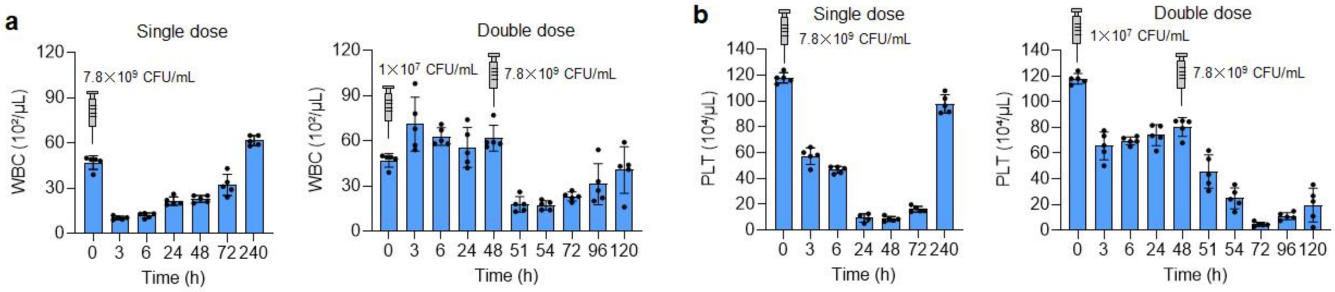

Supplementary Figure S14. Complete blood counts of (a) white blood cells (WBC) and (b) platelets (PLT) in BALB/c-nu/nu mice after a single (left) or double dose (right) of AUN, using the same dose levels as in the cytokine assays. Data are represented as mean ± standard error of the mean; n = 5 biologically independent mice.

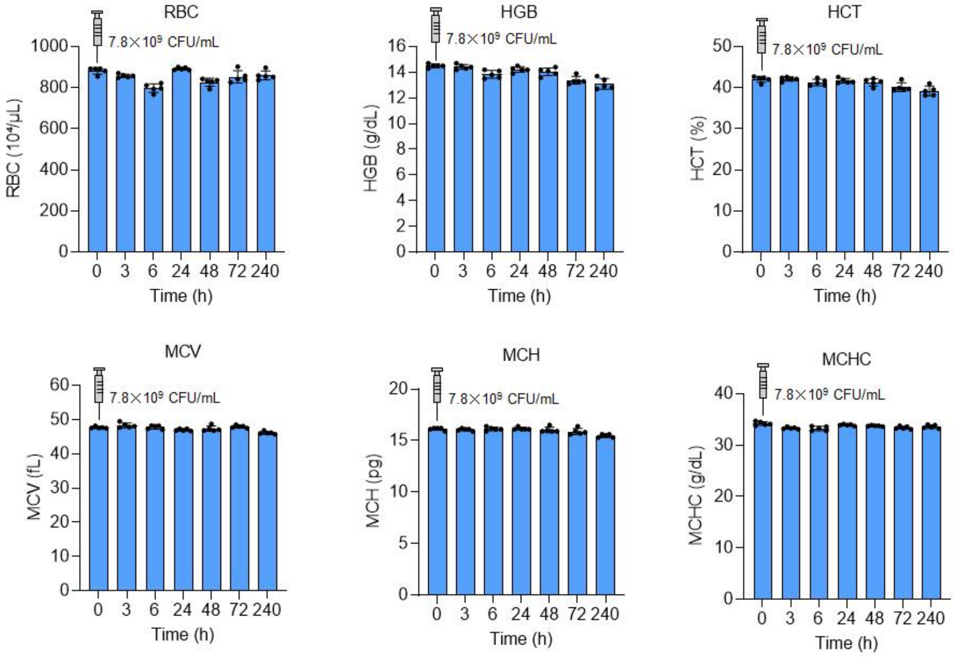

Supplementary Figure S15. Complete blood counts of red blood cell (RBC), haemoglobin (HGB), haematocrit (HCT), mean corpuscular volume (MCV), mean corpuscular haemoglobin (MCH), and mean corpuscular haemoglobin concentration (MCHC) in BALB/c-nu/nu mice after a single dose of AUN. Data are represented as mean ± standard error of the mean; n = 5 biologically independent mice.

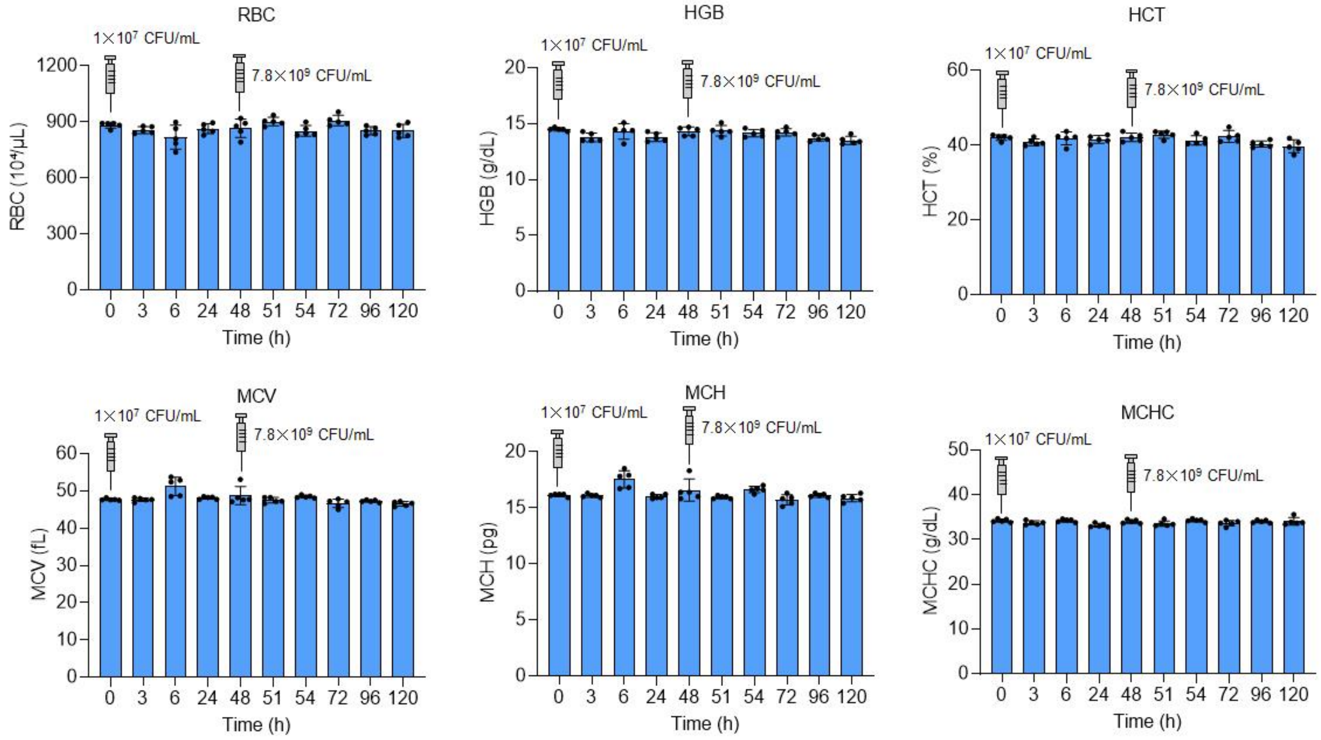

Supplementary Figure S16. Red blood cell count (RBC), haemoglobin concentration (HGB), haematocrit (HCT), mean corpuscular volume (MCV), mean corpuscular haemoglobin (MCH), and mean corpuscular haemoglobin concentration (MCHC) in BALB/c-nu/nu mice after double dose of AUN. Data are represented as mean ± standard error of the mean; n = 5 biologically independent mice.

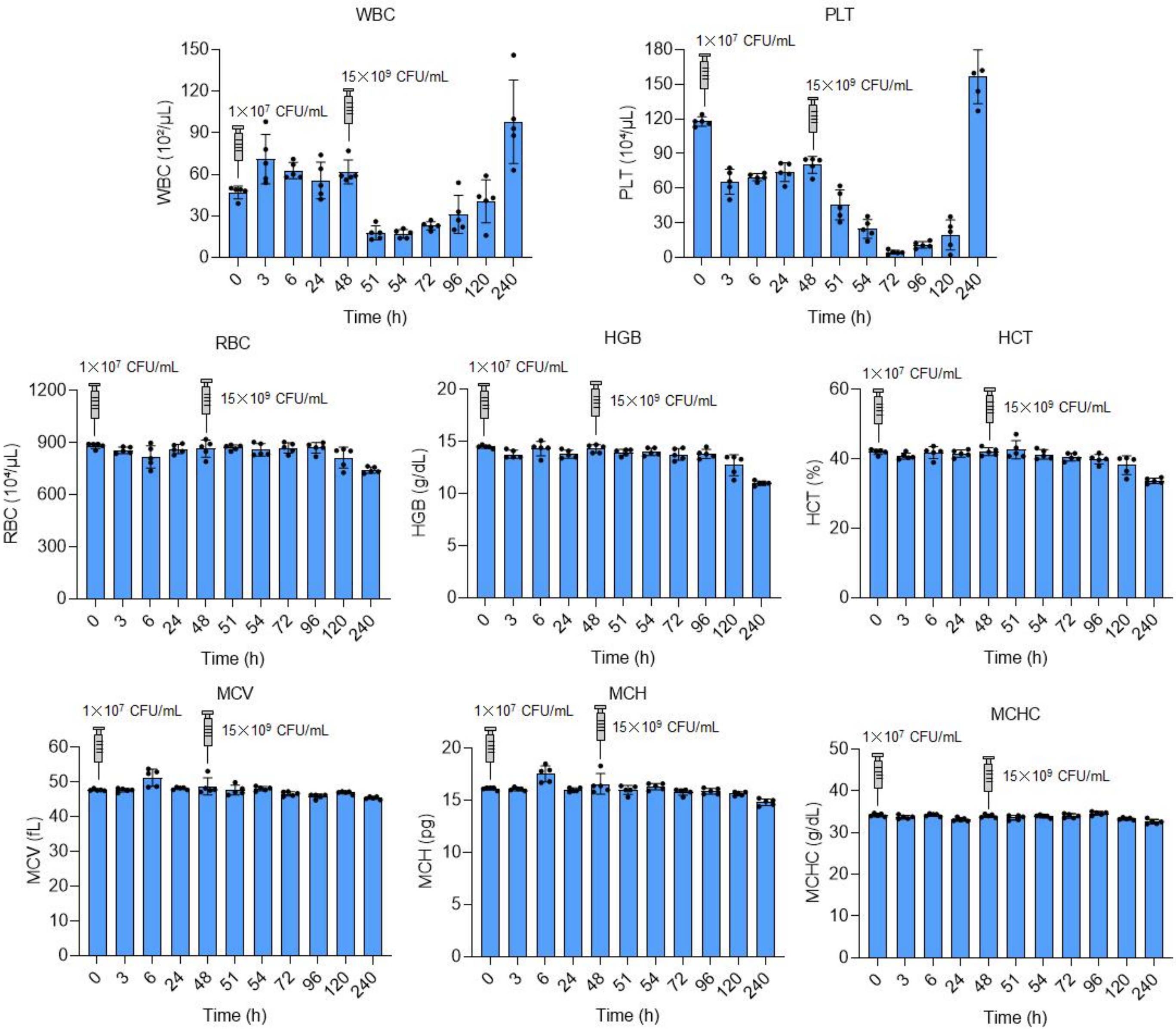

Supplementary Figure S17. White blood cell count (WBC), platelet count (PLT), red blood cell count (RBC), haemoglobin concentration (HGB), haematocrit (HCT), mean corpuscular volume (MCV), mean corpuscular haemoglobin (MCH), and mean corpuscular haemoglobin concentration (MCHC) in BALB/c-nu/nu mice after double dose of AUN. Data are represented as mean ± standard error of the mean; n = 5 biologically independent mice.

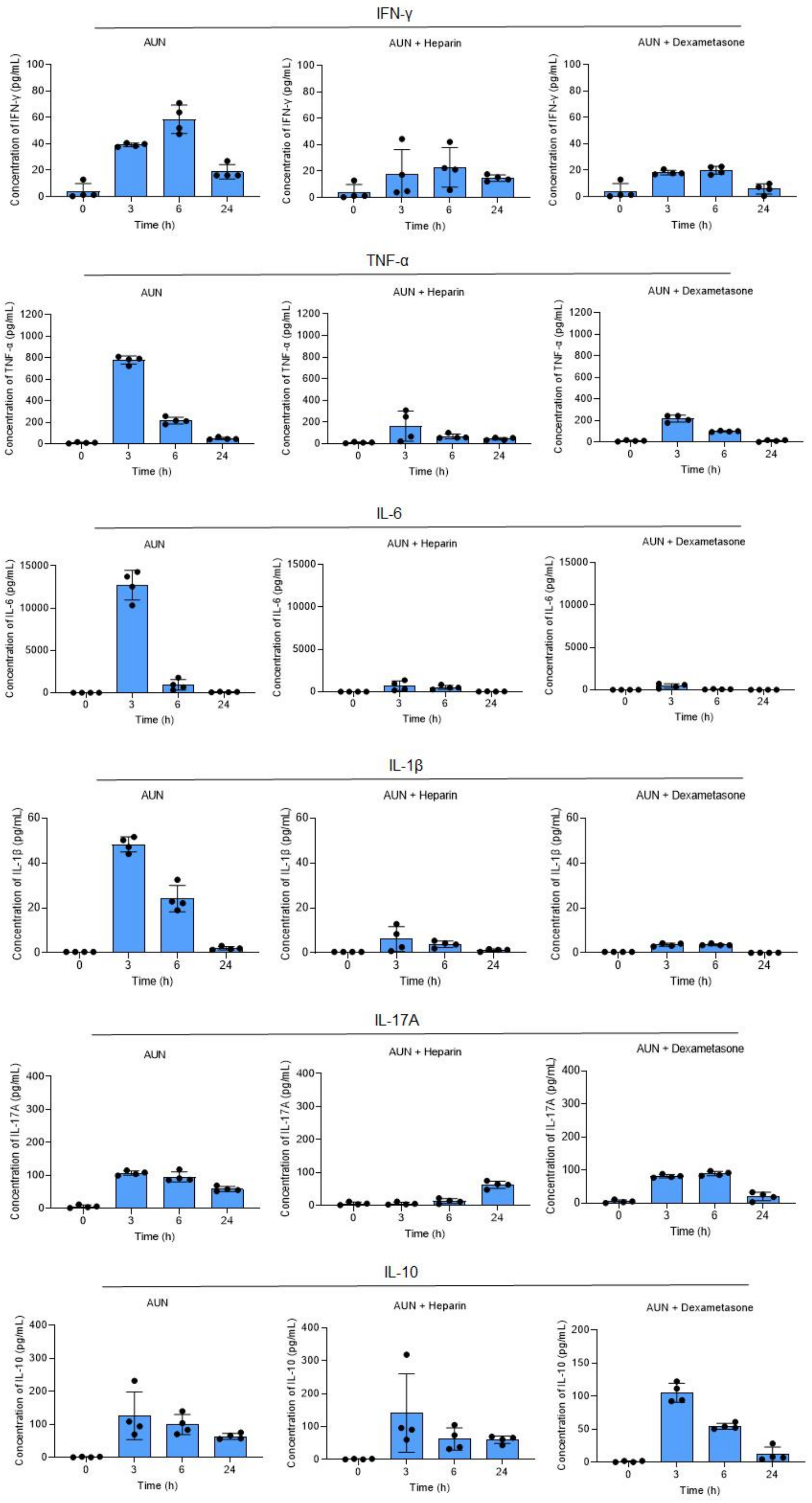

Supplementary Figure S18. Cytokine assays in blood of BALB/c-nu/nu mice after the injection of high dose of AUN (200 μL, 15 × 10^9 CFU/mL) with and without heparin or dexamethasone premedication for measurement of (a) IFN-γ, (b) TNF-α, (c) IL-6, (d) IL-1β, (e) IL-17A, and (f) IL-10. Data are represented as mean ± standard error of the mean; n = 4 biologically independent mice.

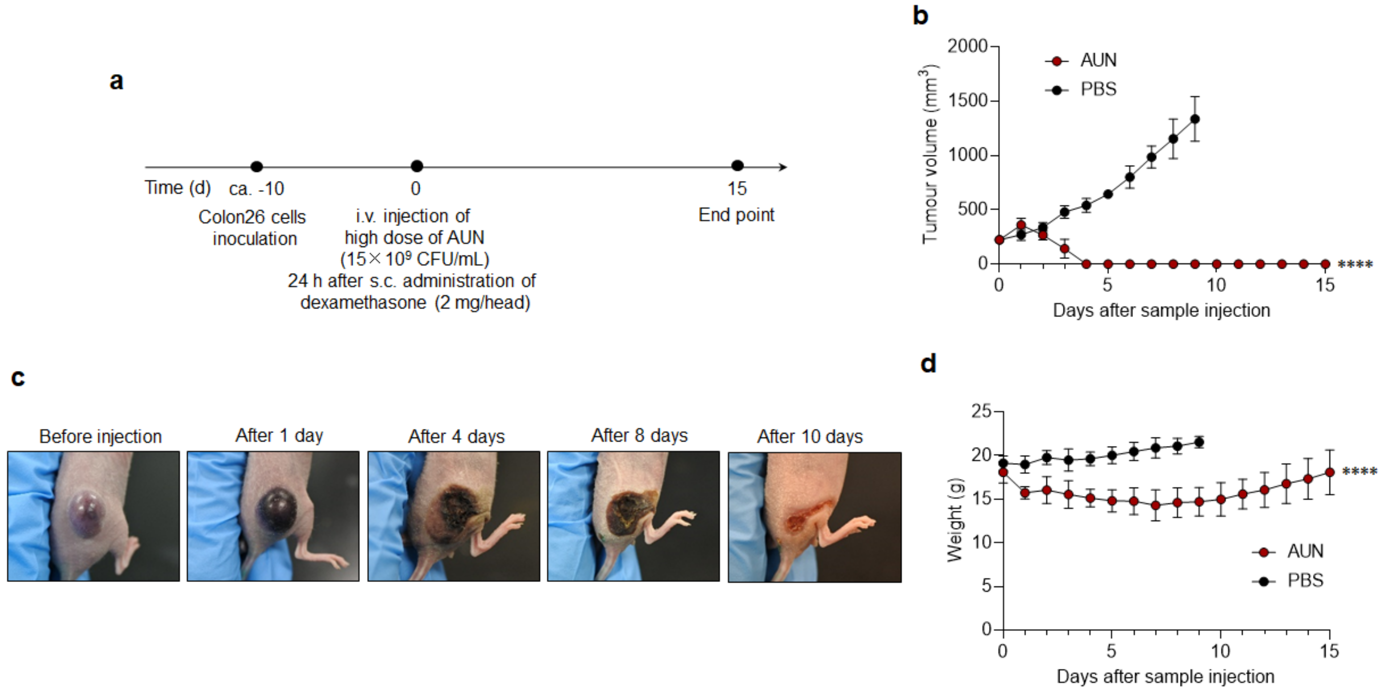

Supplementary Figure S19. Antitumour efficacy of AUN in Colon26 tumour–bearing BALB/c-nu/nu mice previously treated with dexamethasone. (a) Time course of establishment and treatment of mice. After tumour establishment, mice were intravenously (i.v.) injected with heparin and AUN in accordance with the time course. (b) *In vivo* anticancer effect of AUN in Colon26 tumour–bearing BALB/c-nu/nu mice after administration with dexamethasone (2 mg/head). The suspension of AUN (15×10^9 CFU/mL) was i.v. injected once. Statistical significance at the endpoint was calculated by comparison with the PBS group. Data are represented as the mean ± standard errors of the mean (SEM); n = 5 biologically independent mice. ****, $p < 0.0001$, by Student's two-sided *t*-test. (c) Images of mice after the treatment of AUN. (d) Body weight of mice was measured daily after antiplatelet drug treatment followed by a single dose of AUN (15×10^9 CFU/mL). Statistical significance at the endpoint was calculated by comparison with the PBS group. Data are represented as the mean ± SEM; n = 5 biologically independent mice. ****, $p < 0.0001$, by Student's two-sided *t*-test.

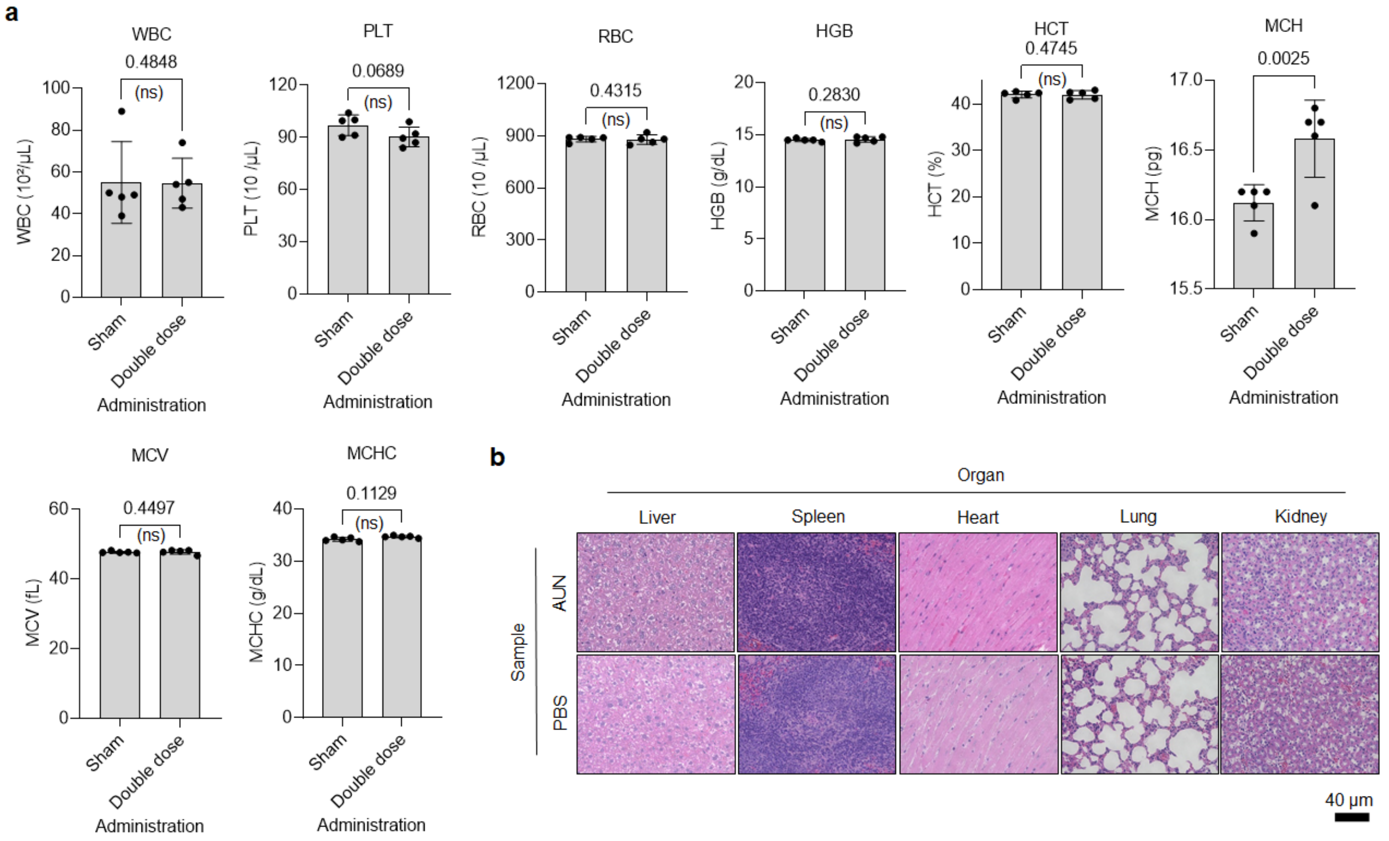

Supplementary Figure S20. Biocompatibility test of two doses of AUN in BALB/c-nu/nu mice. (a) Complete blood counts of mice 14 days after the second of two doses of AUN or a single dose of PBS. The AUN concentrations of the 1st and 2nd doses were 1×10^7 CFU/mL and 15×10^9 CFU/mL, respectively. Data are represented as mean ± standard error of the mean; n = 5 independent experiments. WBC, white blood cell count; PLT, platelet count; RBC, red blood cell count; HGB, haemoglobin concentration; HCT, haematocrit; MCV, mean corpuscular volume; MCH, mean corpuscular haemoglobin; MCHC, mean corpuscular haemoglobin concentration; ns, not significant; *p*-values were obtained using Student's two-sided *t*-test. The obtained values are within the normal limits for BALB/c-nu/nu mice as reported by the vendor. (b) Haematoxylin and eosin (H&E) staining in conventional organs sectioned 14 days after two doses of AUN, at the same concentrations used in (a), or a single dose of PBS.

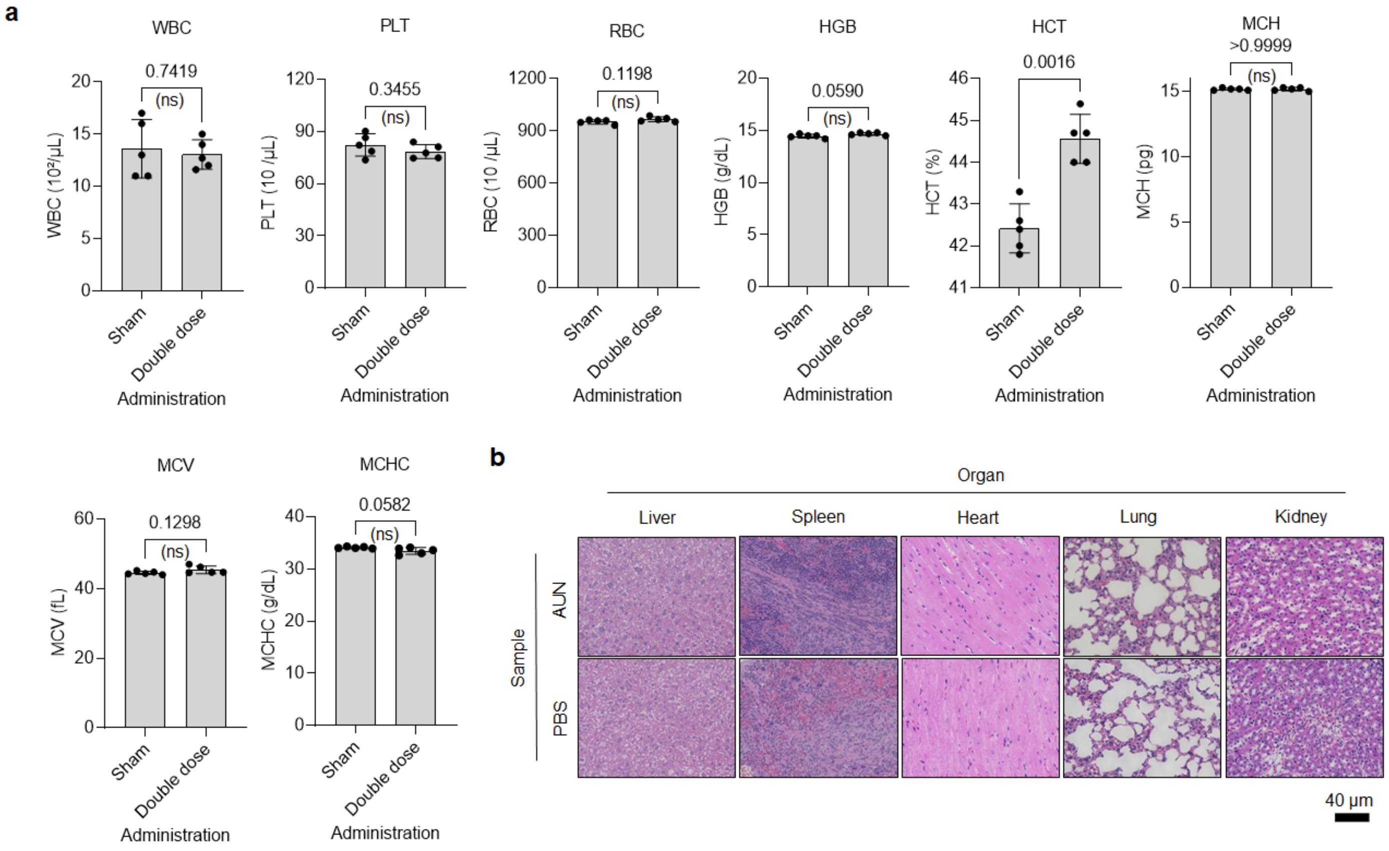

Supplementary Figure S21. Biocompatibility test of two doses of AUN for severe combined immunodeficient (SCID) mice. (a) Complete blood counts of the mice 14 days after two doses of AUN or a single dose of PBS. The AUN concentrations of the 1st and 2nd doses were 1×10^7 CFU/mL and 7×10^9 CFU/mL, respectively. Data are represented as mean ± standard error of the mean; n = 5 independent experiments. WBC, white blood cell; PLT, platelet; RBC, red blood cell; HGB, haemoglobin; HCT, haematocrit; MCV, mean corpuscular volume; MCH, mean corpuscular haemoglobin; MCHC, mean corpuscular haemoglobin concentration; ns, not significant; *p*-values were obtained using Student's two-sided *t*-test. The obtained values are within the normal limits of complete blood counts of a SCID mouse given in the manufacturer's technical information. (b) Haematoxylin and eosin (H&E) staining in conventional organs sectioned 14 days after two doses of AUN, at the same concentrations used in (a), or a single administration of PBS.

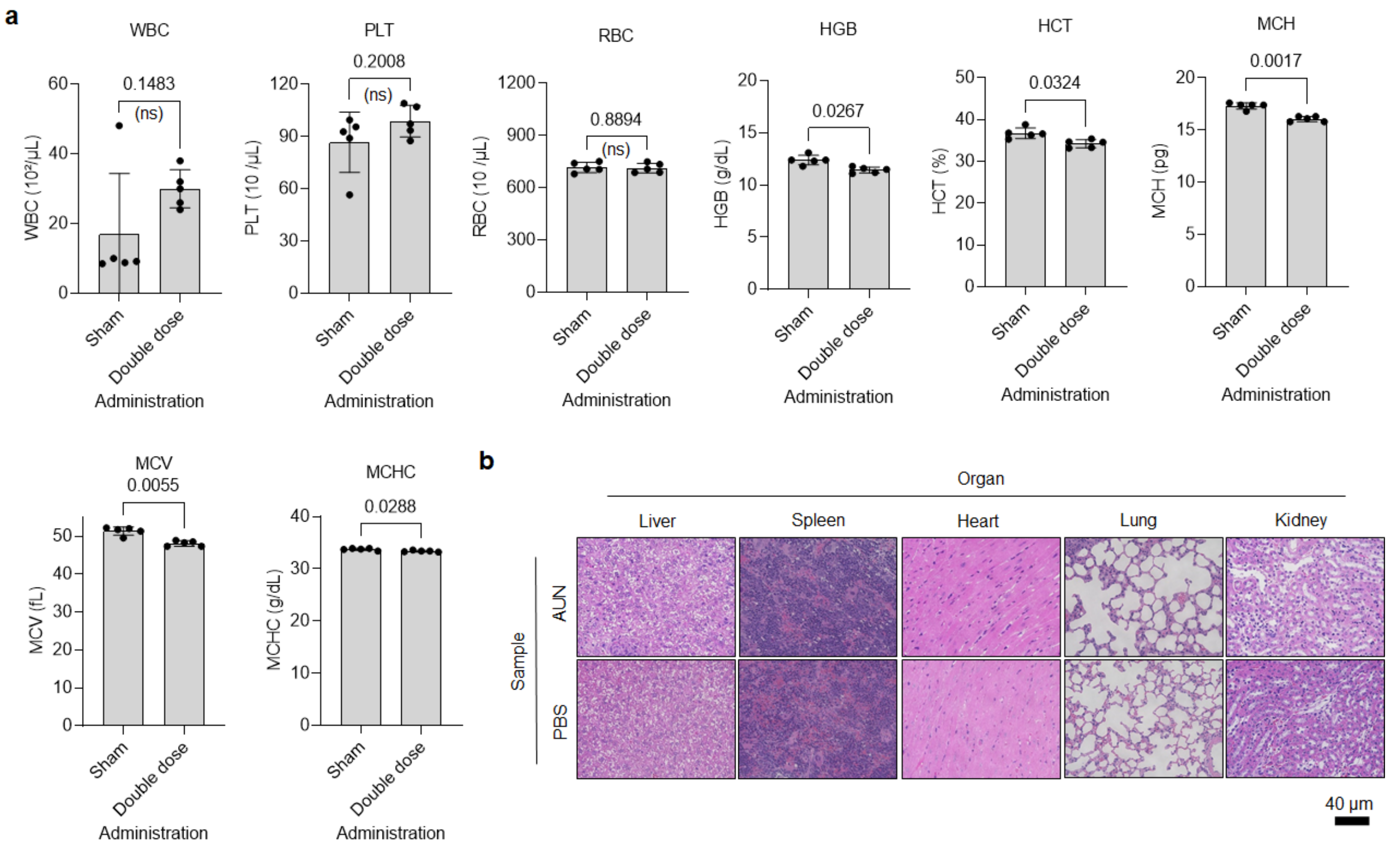

Supplementary Figure S22. Biocompatibility test of a double dose of AUN for non-obese diabetic severe combined immunodeficient (NOD-SCID) mice. (a) Complete blood counts of the mice 14 days after two doses of AUN or a single dose of PBS. The AUN concentrations of the 1st and 2nd doses were 1×10^8 CFU/mL and 15×10^9 CFU/mL, respectively. Data are represented as mean ± standard error of the mean; n = 5 independent experiments. ns, not significant; *p*-values were obtained using Student's two-sided *t*-test. The obtained values are within the normal limits of complete blood counts of NOD-SCID mice given in the manufacturer's technical information. WBC, white blood cells; PLT, platelets; RBC, red blood cells; HGB, haemoglobin; HCT, haematocrit; MCH, mean corpuscular haemoglobin; MCV, mean corpuscular volume; MCHC, mean corpuscular haemoglobin concentration. (b) Haematoxylin and eosin (H&E) staining in conventional organs sectioned 14 days after two doses of AUN or a single administration of PBS. The AUN concentrations of the 1st and 2nd doses were 1×10^8 CFU/mL and 15×10^9 CFU/mL, respectively.

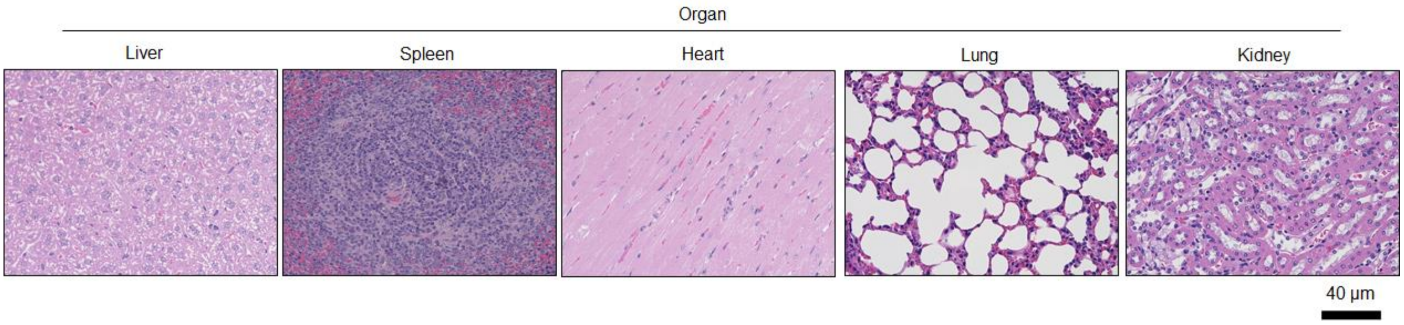

Supplementary Figure S23. Haematoxylin and eosin (H&E) staining in conventional organs sectioned 150 days after achieving CR of tumours by two doses of AUN. The AUN concentrations of the 1st and 2nd doses were 1×10^8 CFU/mL and 15×10^9 CFU/mL, respectively.

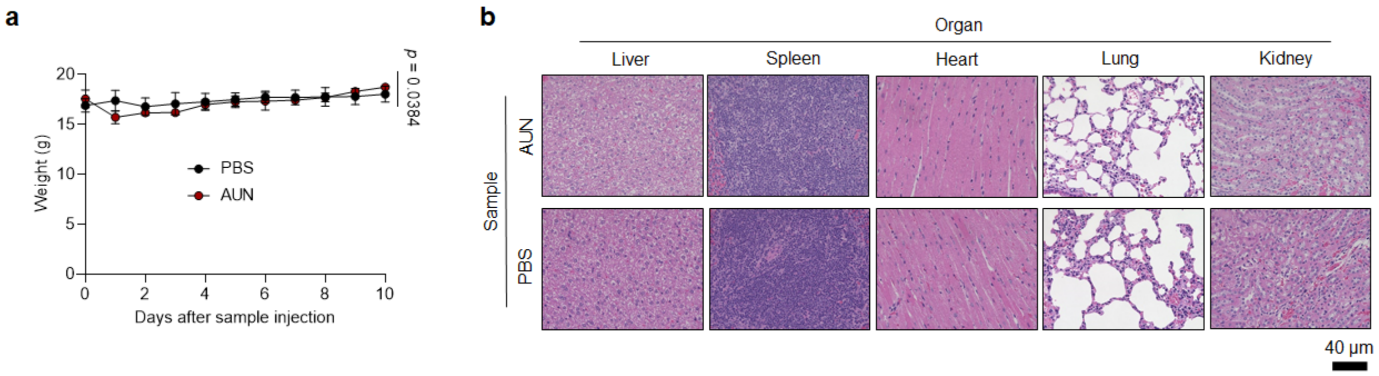

Supplementary Figure S24. Safety tests of AUN using lower-limb ischemia model mice. (a) Body weight of mice was measured daily after the treatment followed by a single dose of AUN (200 μL, 5 × 10^9 CFU/mL) or PBS (200 μL). Statistical significance was calculated by comparison with the PBS group. Data are represented as mean ± standard error of the mean; n = 5 independent experiments. ns, not significant (p = 0.4571), by two-way ANOVA. (b) Haematoxylin and eosin (H&E) staining in vital organs of the lower-limb ischemia model mice sectioned 30 days after single dose of AUN (200 μL) or a single dose of PBS (200 μL). The AUN concentration was 5 × 10^9 CFU/mL.

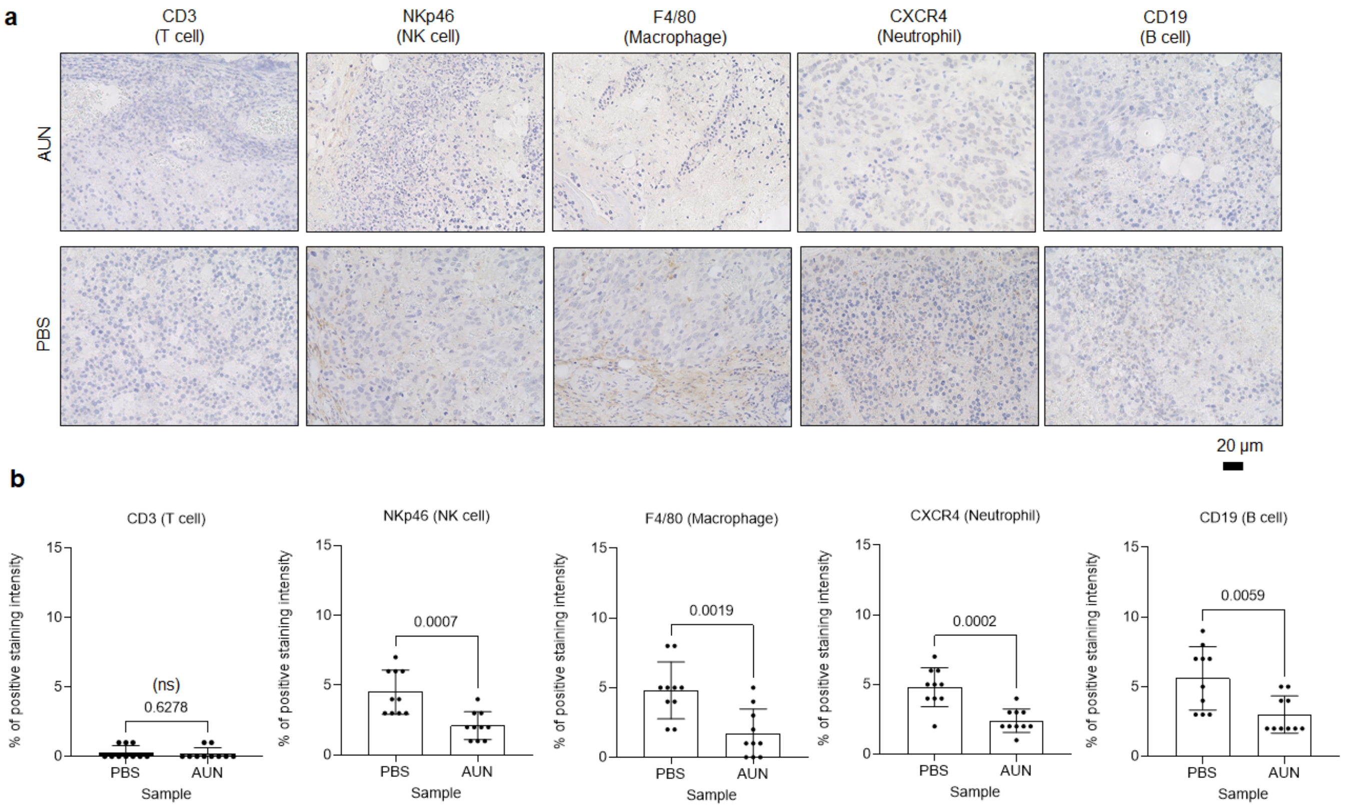

Supplementary Figure S25. Immunological activity in the Colon26 tumours of BALB/c-nu/nu mice after treatment with AUN, measured by the immunohistochemical staining method. (a) Tumour tissues were collected from Colon26 tumour-bearing BALB/c-nu/nu mice on day 1 after a single dose administration with AUN and PBS and stained for CD3, NKp46, F4/80, CXCR4, and CD19. (b) Statistical analyses of cells positive for CD3, NKp46, F4/80, CXCR4, and CD19 positive. Data are represented as mean ± standard error of the mean; n = 10 independent areas (region of interest) in each tumour tissue collected from the groups of mice on day 1 after a single dose administration with AUN or PBS. Statistical significance was calculated in comparison with the PBS group. ns, not significant; p-values were obtained by Student's two-sided t-test.

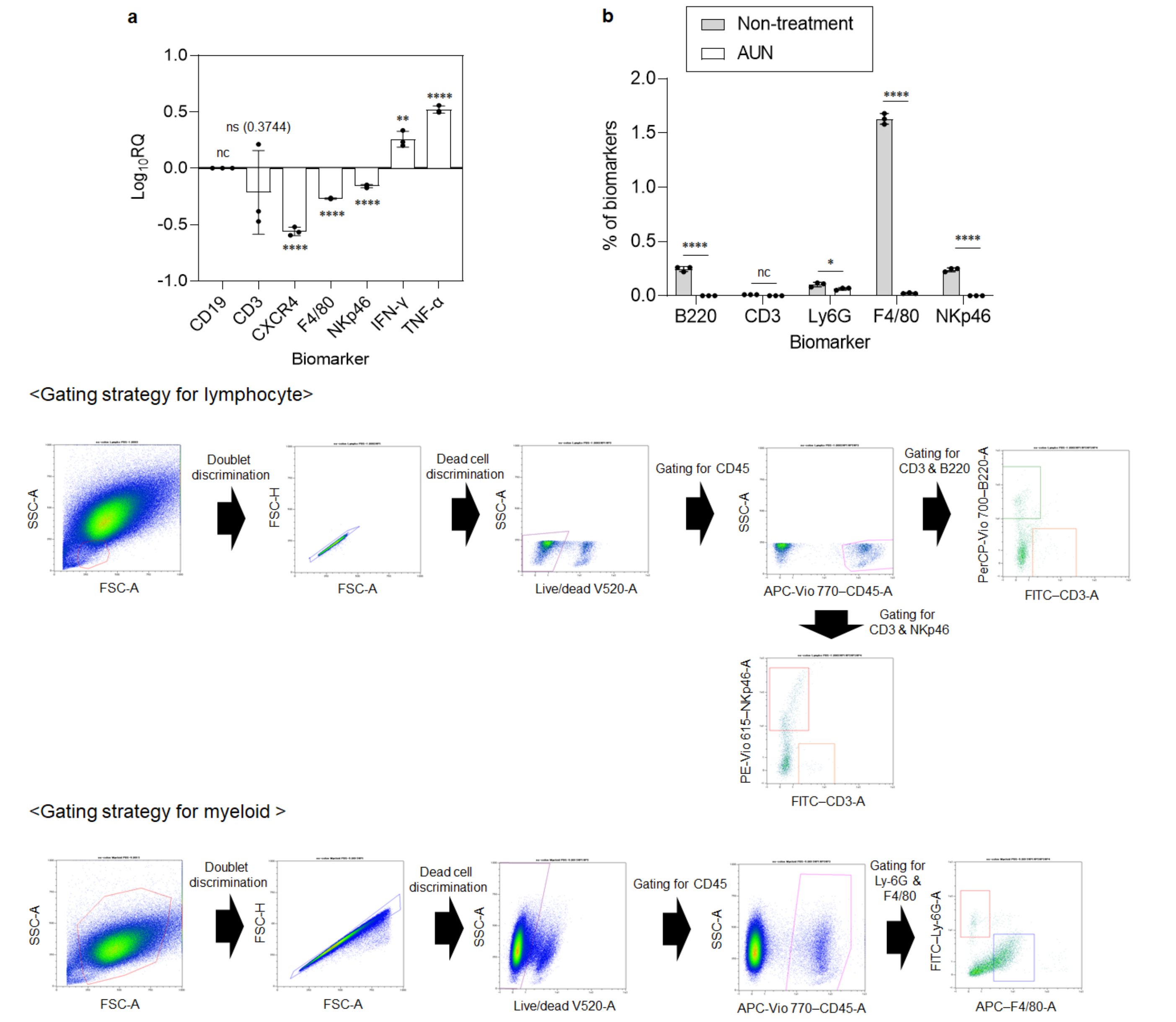

Supplementary Figure S26. Immunological activity in the Colon26 tumours of BALB/c-nu/nu mice after a single dose administration with AUN, measured by quantitative polymerase chain reaction (qPCR) and flow cytometry. (a) Quantification by qPCR of markers and cytokines related to immune cells 24 h after intravenous administration with AUN (200 µL, 7.8×10^9 CFU/mL). The mRNA expression of CD19, CD3, CXCR4, F4/80, NKp46, and cytokines (IFN-γ and TNF-α) are shown as fold change at log 10 relative quantification (RQ) relative to the control group (non-treatment). GAPDH gene expression was used as an internal control. Statistical significance was calculated in comparison with the non-treatment group. nc, not calculable (statistical testing could not be performed because all values in both groups were identical), ns, not significant, **, $p = 0.0032$, and ****, $p < 0.0001$, by Student's two-sided *t*-test. Data are represented as mean ± standard error of the mean (SEM); n = 3 independent tumour samples. (b) Flow cytometry analyses of the expression of cells positive for B220, CD3, Ly6G, F4/80, and NKp46 in tumours 24 h after intravenous injection of AUN. Data are represented as the mean ± SEM; n = 3 independent tumour tissues. Statistical significance was calculated in comparison with the non-treatment group. nc, not calculable (statistical testing could not be performed because all values in both groups were identical), ns, not significant, *, $p = 0.0437$, and ***, $p < 0.001$, by Student's two-sided *t*-test. The gating strategy plots show a representative example identifying CD3+ cells.

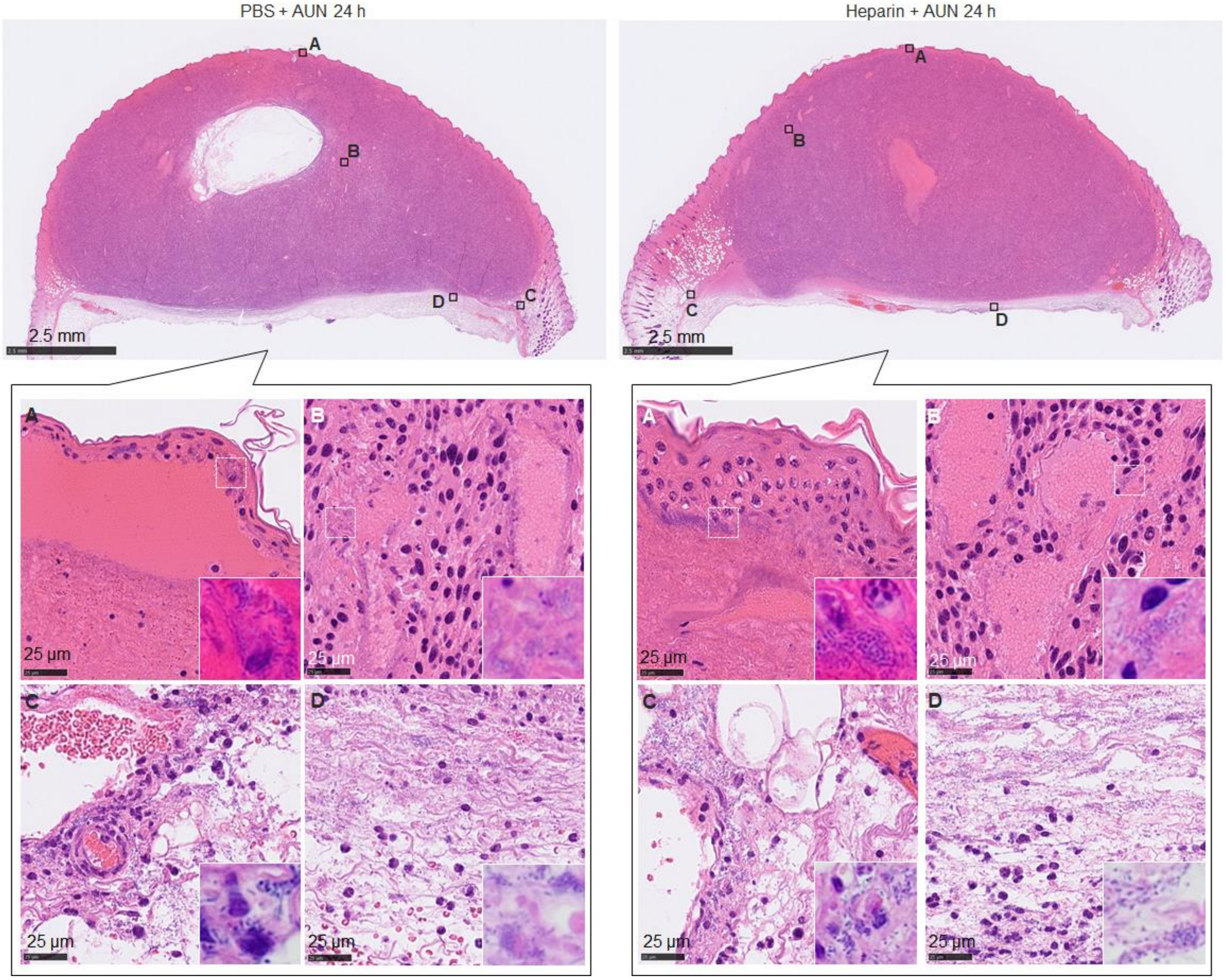

Supplementary Figure S27. Bacterial location in tumours of AUN-treated mice after administration with and without heparin. H&E–stained tumour tissues collected from different groups of mice at 24 h after respective treatments. AUN (200 µL, 5×10^9 CFU/mL) was i.v. injected to Colon26-bearing BALB/c-nu/nu mice (n = 3 biologically independent mice) with or without administration of heparin (5 mg/head) for 1 h. Bacterial growth is mainly observed just below the basal layer of the epidermis (A), in the tumour's internal vessel wall (B), in and around the tumour's blood vessel wall (C), and in the connective tissue at the base of the tumour (D).

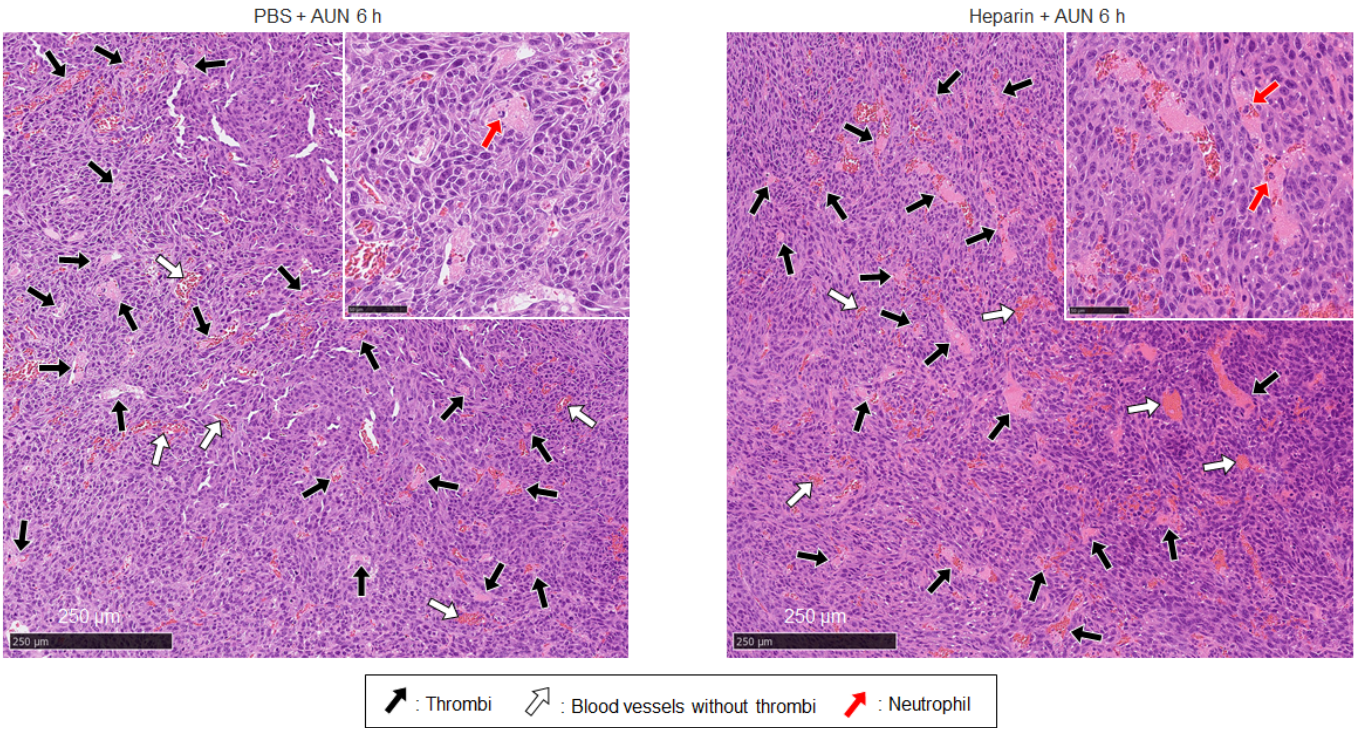

Supplementary Figure S28. Distribution of intratumoural thrombi 6 h after administration of AUN (n = 3 independent experiments).

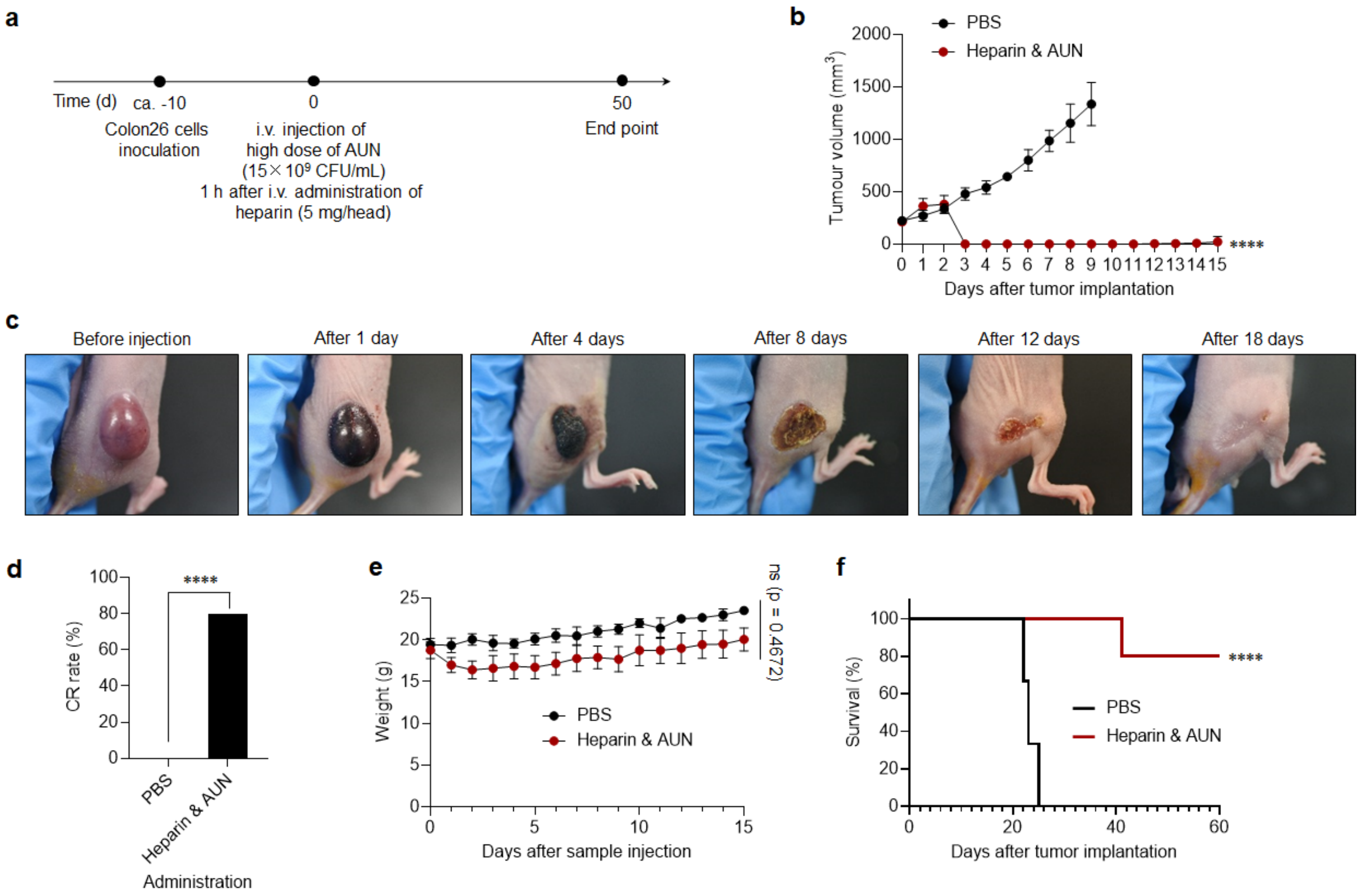

Supplementary Figure S29. Antitumour efficacy of AUN in Colon26 tumour–bearing BALB/c-nu/nu mice previously treated with heparin. (a) Time course of establishment and treatment of mice. After tumour establishment, mice were intravenously (i.v.) injected with heparin and AUN in accordance with the time course. (b) *In vivo* anticancer effect of AUN in mice after administration with heparin (5 mg/head). The suspension of AUN (15×10^9 CFU/mL) was i.v. injected once. The control experiment (a single i.v. dose of PBS) was also performed. Data are represented as the mean ± standard errors of the mean (SEM); n = 5 biologically independent mice. Statistical significance at the endpoint was calculated by comparison with the PBS group. ****, $p < 0.0001$, by Student's two-sided *t*-test. (c) Images of mice after the treatment. (d) CR rate of mice (n = 5 biologically independent mice) 60 days after heparin treatment followed by AUN or PBS injection. ****, $p < 0.0001$, by Student's t two-sided test. (e) Body weight of mice measured daily after heparin treatment followed by a single dose of AUN (15×10^9 CFU/mL) or PBS. Data are represented as mean ± SEM; n = 5 independent experiments. Statistical significance was calculated by comparison with the PBS group. ns, not significant; *p*-value was obtained using two-way ANOVA. (f) Kaplan–Meier survival curves (n = 5 biologically independent mice) for 30 days after tumour implantation. Statistical significance at the endpoint was calculated by comparison with the PBS group. ****, $p < 0.0001$, by log-rank (Mantel-Cox) test.

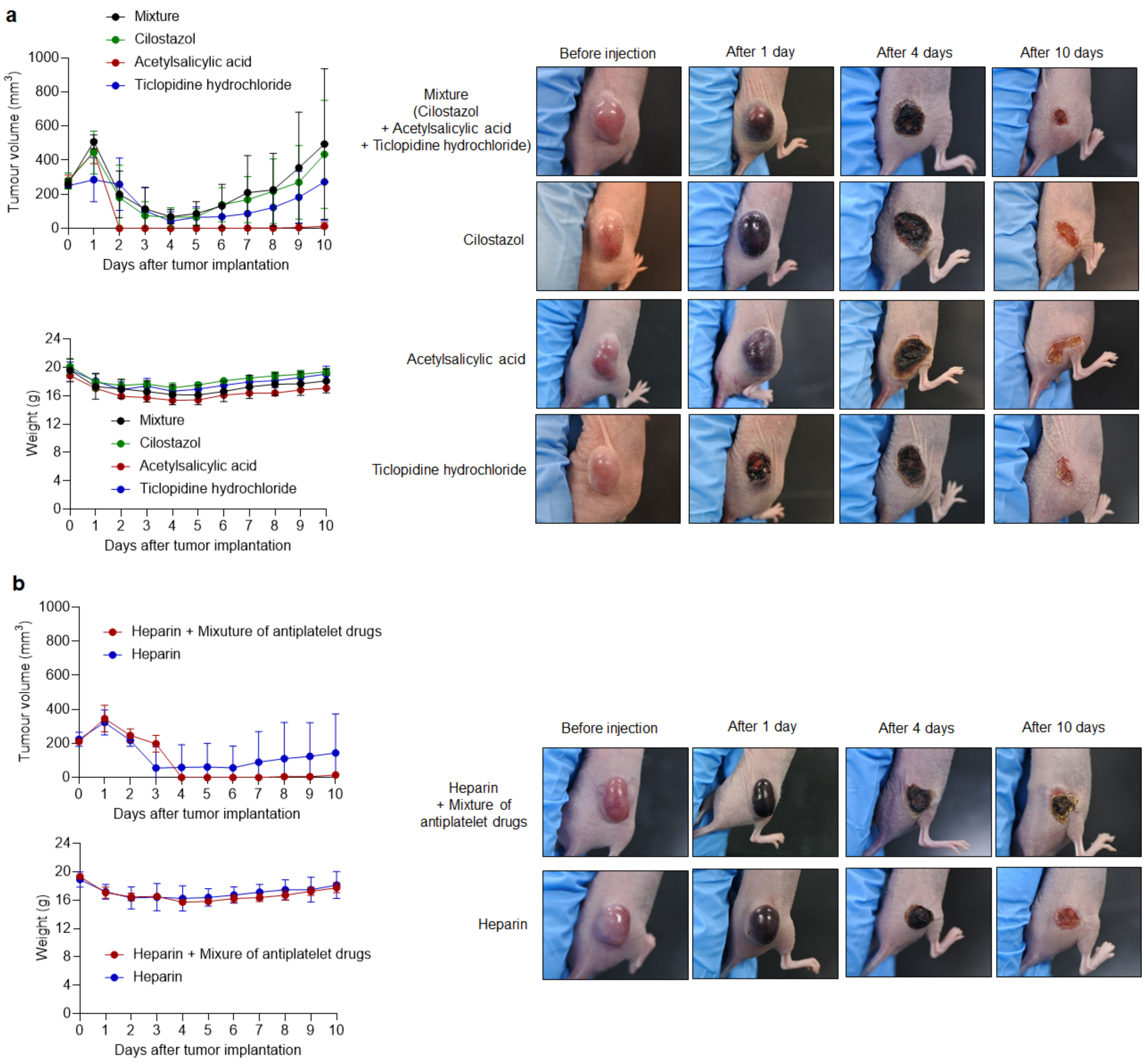

Supplementary Figure S30. Antitumour efficacy and tumour-specific thrombus formation by AUN with administration of various antiplatelet drugs or the combination of antiplatelet drugs and heparin. (a) *In vivo* anticancer effect of AUN in Colon26 tumour–bearing BALB/c-nu/nu mice after administration with an antiplatelet drug (1 mg/head) or the mixture of three antiplatelet drugs (each drug dose = 1 mg/head; total dose = 3 mg/head). The suspension of AUN (7.8 × 10^9 CFU/mL) was i.v. injected once. Data are represented as the mean ± standard errors of the mean (SEM); n = 5 biologically independent mice. Body weight of mice was measured daily after antiplatelet drug treatment followed by a single dose of AUN (7.8 × 10^9 CFU/mL). (b) *In vivo* anticancer effect of AUN in Colon26 tumour–bearing BALB/c-nu/nu mice after administration with heparin (5 mg/head) and the mixture of three antiplatelet drugs (each drug dose = 1 mg/head; total drug dose = 3 mg/head). The suspension of AUN (7.8 × 10^9 CFU/mL) was i.v. injected once. Data are represented as the mean ± SEM; n = 5 biologically independent mice. Body weight of mice was measured daily after heparin and antiplatelet drugs treatment followed by a single dose of AUN (7.8 × 10^9 CFU/mL).

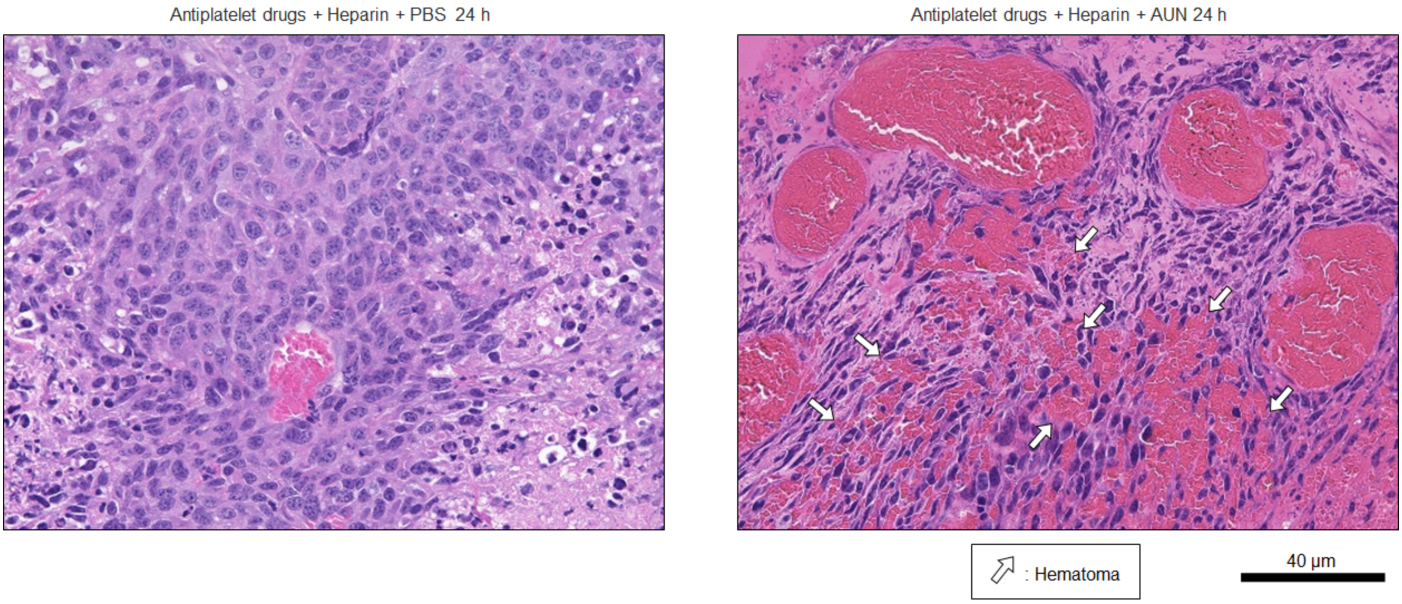

Supplementary Figure S31. Distribution of intratumoural hematoma in Colon26 tumour–bearing BALB/c-nu/nu (n = 3 biologically independent mice) 24 h after single-dose administration of PBS (placebo) (200 µL) (left) or AUN (200 µL, 5 × 10^9 CFU/mL) (right). The mixture of three antiplatelet drugs of cilostazol, acetylsalicylic acid, and ticlopidine hydrochloride (each drug dose = 1 mg/head; total dose = 3 mg/head) were administered 24 h before i.v. injection of AUN. Heparin (5 mg/head) was also administered 1 h before injection of AUN.

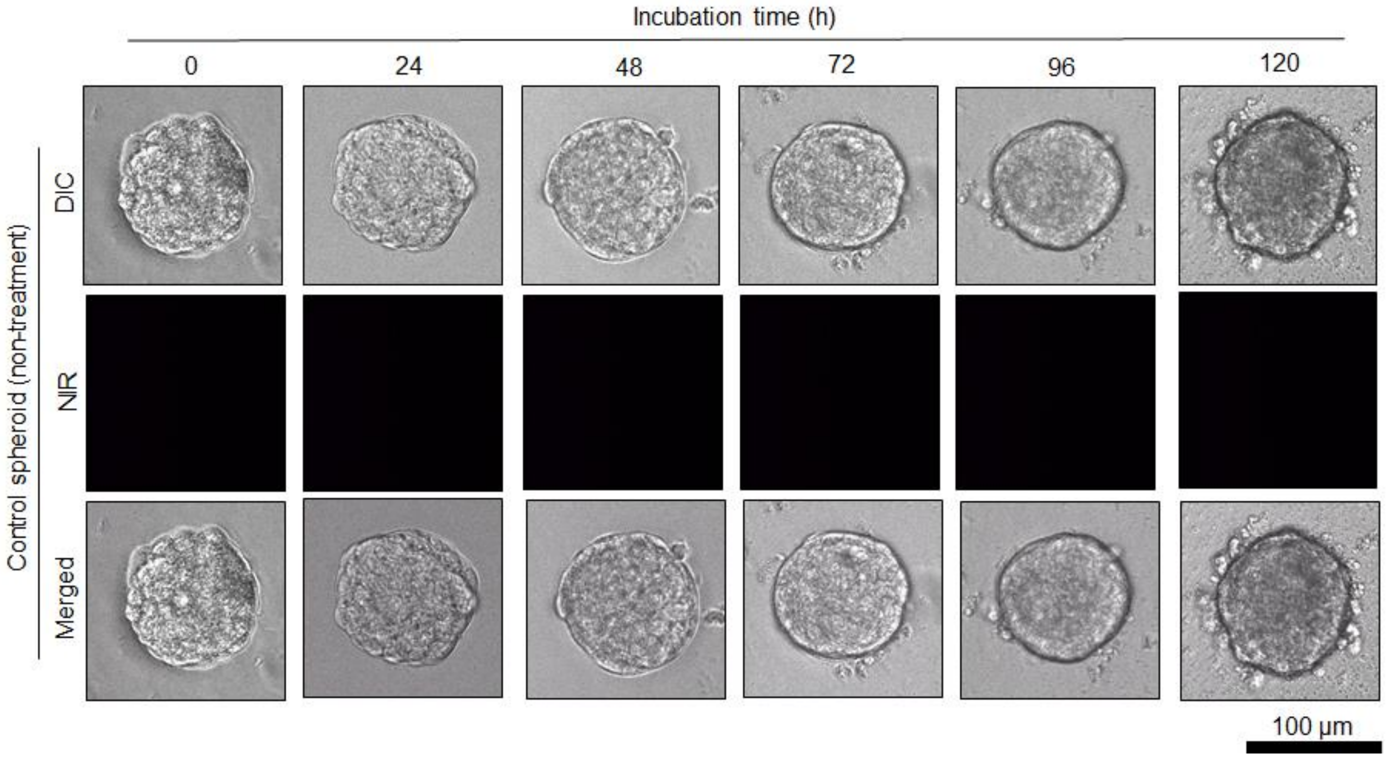

Supplementary Figure S32. Fluorescent imaging of Colon26 cancer spheroids without any treatment, used as a control (n = 3 independent experiments). NIR: Near-infrared, DIC: Differential interference contrast.

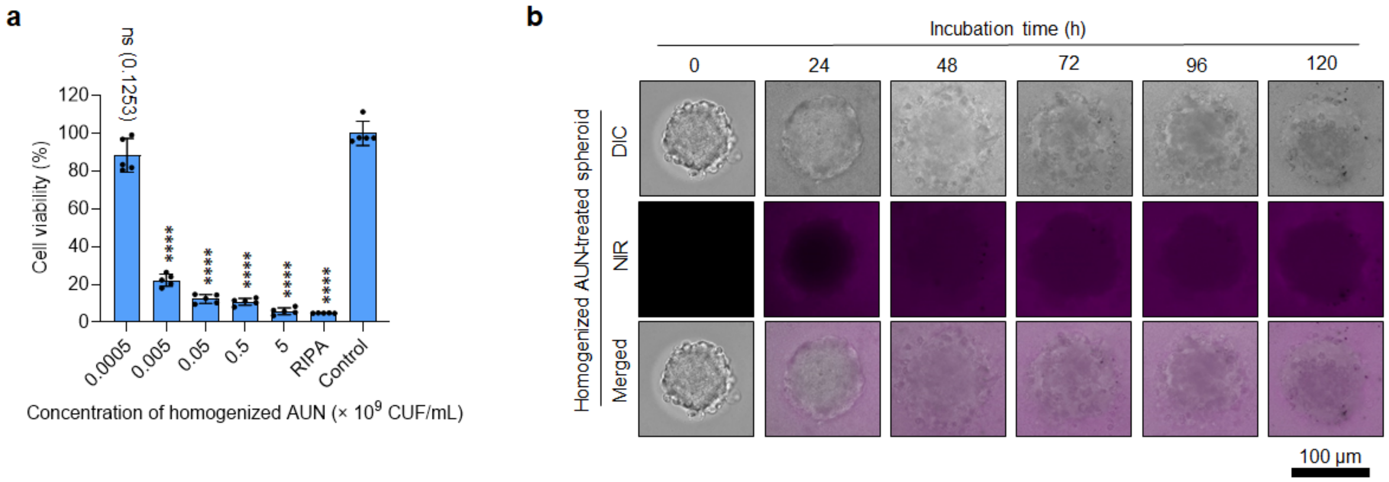

Supplementary Figure S33. Cytotoxicity test and observation of spheroid destruction by homogenized AUN (dead cell). (a) Cytotoxicity of homogenized AUN. The viabilities of Colon26 cells were tested 24 h after treatment with each bacterium at different bacterial concentrations. Data are represented as means ± standard errors of the mean; n = 5 independent experiments. Statistical significance was calculated in comparison with the control group without any sample treatment. ns, not significant and ****, $p < 0.0001$, by Student's two-sided t-test. (b) Optical imaging of Colon26 cancer spheroids with homogenized AUN treatment (n = 3 independent experiments). Concentration of AUN was 5×10^9 CFU/mL.

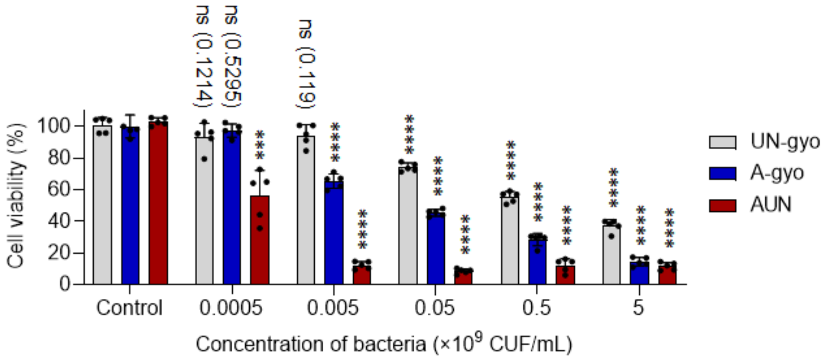

Supplementary Figure S34. Cytotoxicity of UN-gyo, A-gyo, and AUN. The viabilities of Colon26 cells were tested 24 h after treatment with each bacterium at different bacterial concentrations. Data are represented as means ± standard errors of the mean; n = 5 independent experiments. Statistical significance was calculated in comparison with the control group without any sample treatment. ns, not significant, ***, $p = 0.0004$, and ****, $p < 0.0001$, by Student's two-sided t-test.

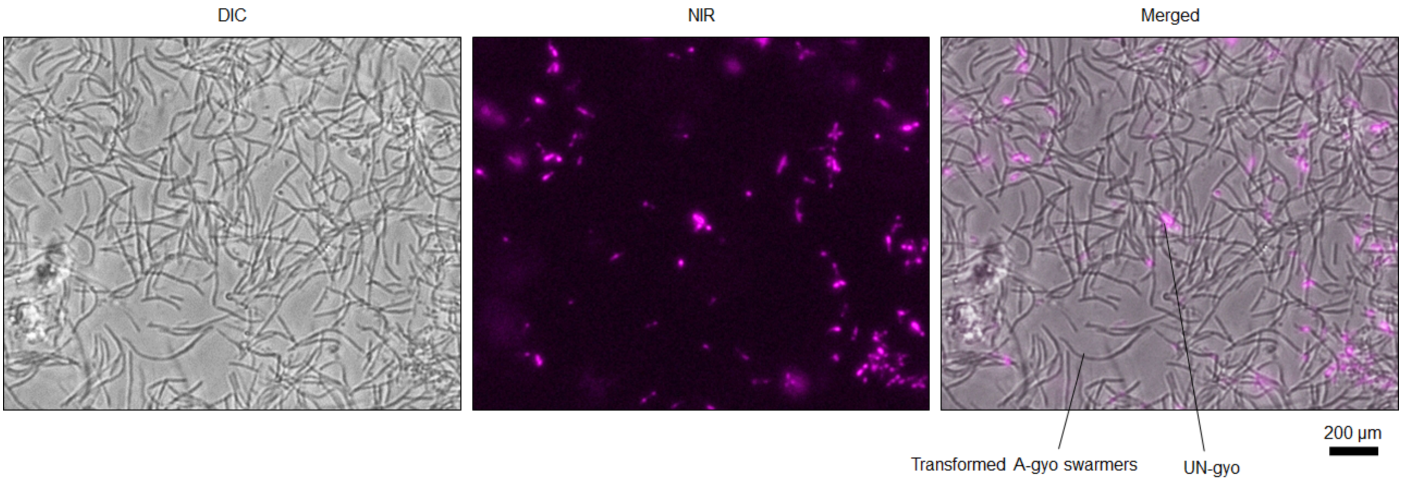

Supplementary Figure S35. Fluorescent images of transformed A-gyo and UN-gyo after co-culturing AUN with Colon26 cells (n = 3 independent experiments). Bacterial concentration was 5×10^8 CFU/mL.

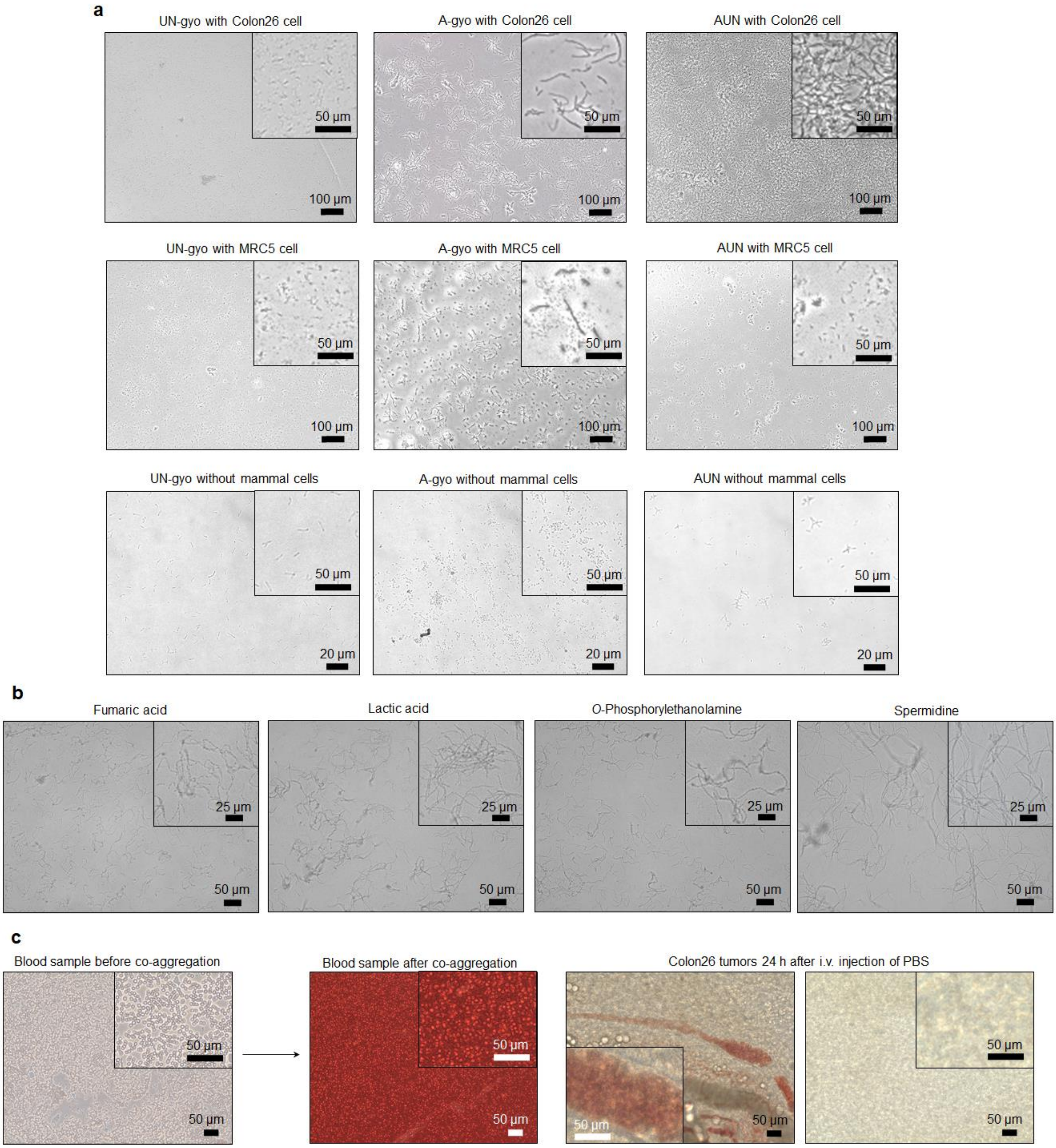

Supplementary Figure S36. Transformation of A-gyo. (a) Optical microscopic images of AUN, A-gyo, and UN-gyo after culturing in a mixed culture medium (cell culture medium:bacterial culture medium = 1:1) at 37 °C in a humidified incubator containing 5% CO_2 for 24 h with Colon26 or MRC5 cells and without mammal cells (n = 3 independent experiments). Bacterial concentration was 5×10^8 CFU/mL. (b) Facilitation of A-gyo transformation by various oncometabolites (fumaric acid, lactic acid, spermidine, and *O*-phosphorylethanolamine). n = 3 independent experiments. (c) Optical microscopic images of blood sample from nude mouse without any treatments (left) and tumour tissues 24 h after i.v. injection of PBS (200 μL) (right). n = 3 independent experiments.

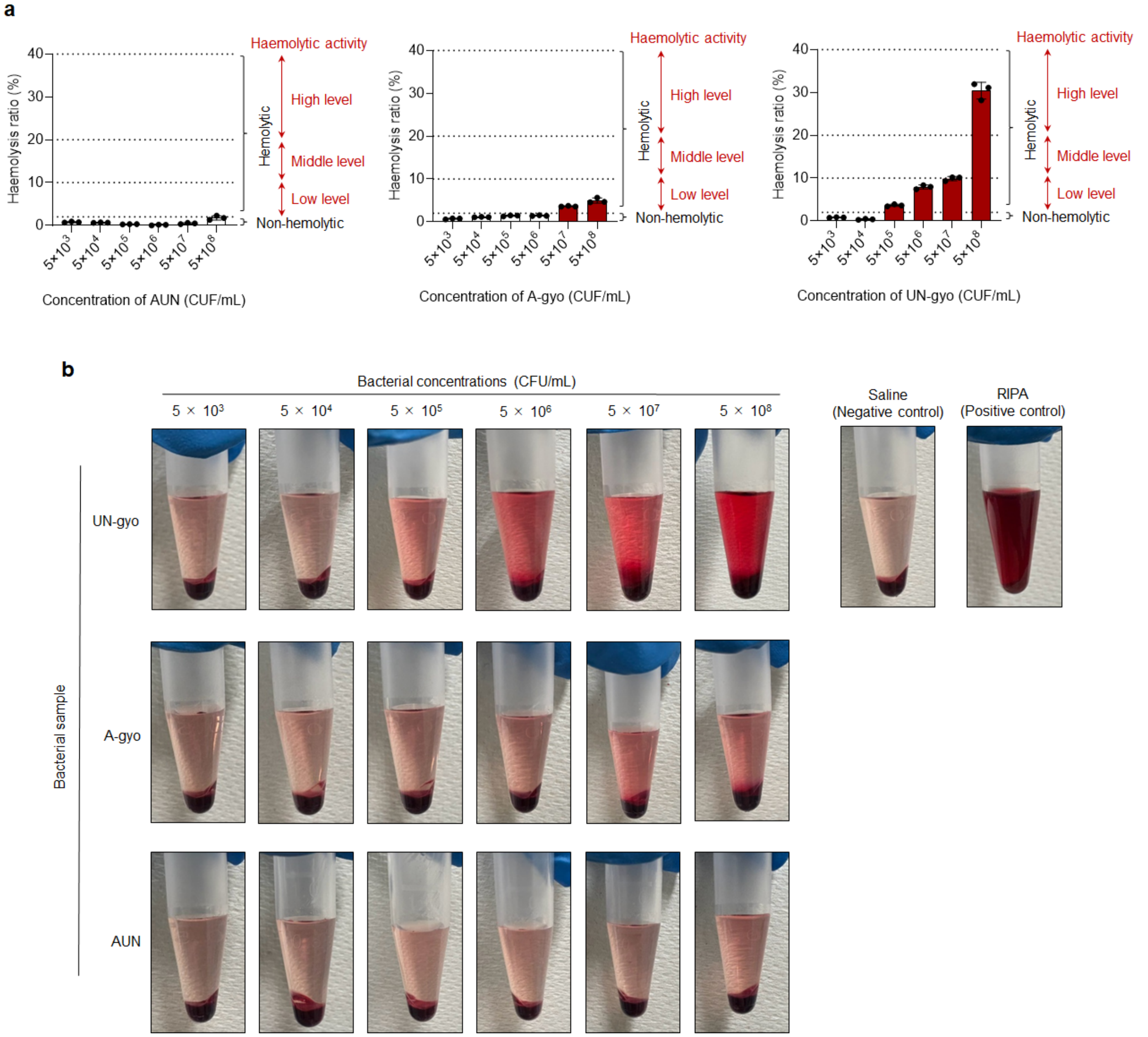

Supplementary Figure S37. Haemolysis activity of AUN. (a) Haemolysis ratios of AUN, A-gyo, and UN-gyo at different concentrations. Saline and RIPA were used as negative and positive controls, respectively. Data are represented as the mean ± standard errors of the mean; n = 3 independent experiments. Red bars represent haemolytic. (b) Photos of each sample after centrifugation (820 × *g*, 5 min, and 4°C).

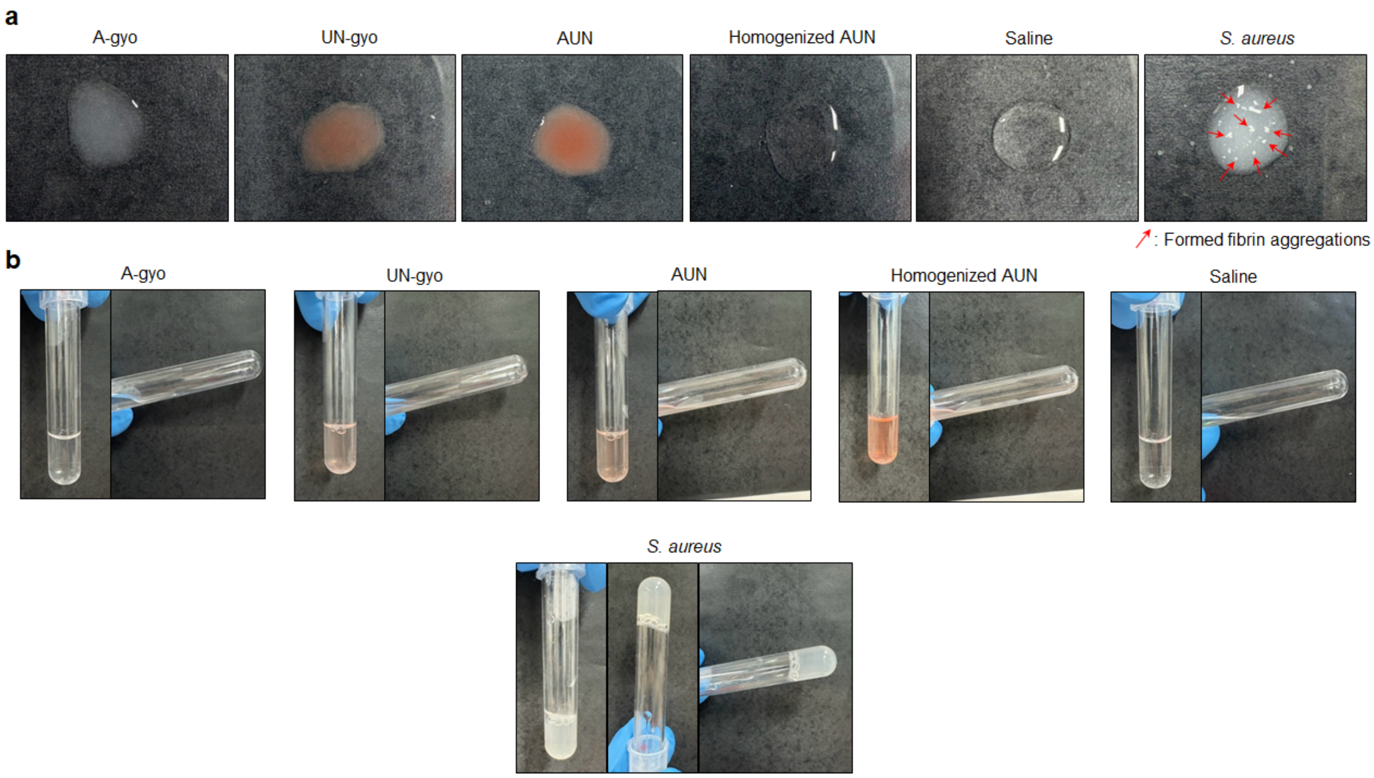

Supplementary Figure S38. Coagulation test. Slide (a) and tube tests (b) of A-gyo, UN-gyo, AUN, homogenized AUN, saline, and *Staphylococcus aureus*. Saline and *S. aureus* were used negative and positive controls, respectively.

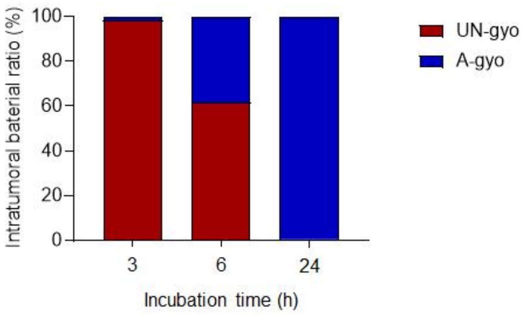

Supplementary Figure S39. Intratumoural bacterial population shift of AUN in Colon26 tumour–bearing BALB/c nude mice 0, 3, 6, and 24 h after intravenous administration of AUN. Data are represented as the average values; n = 3 independent tumour samples.

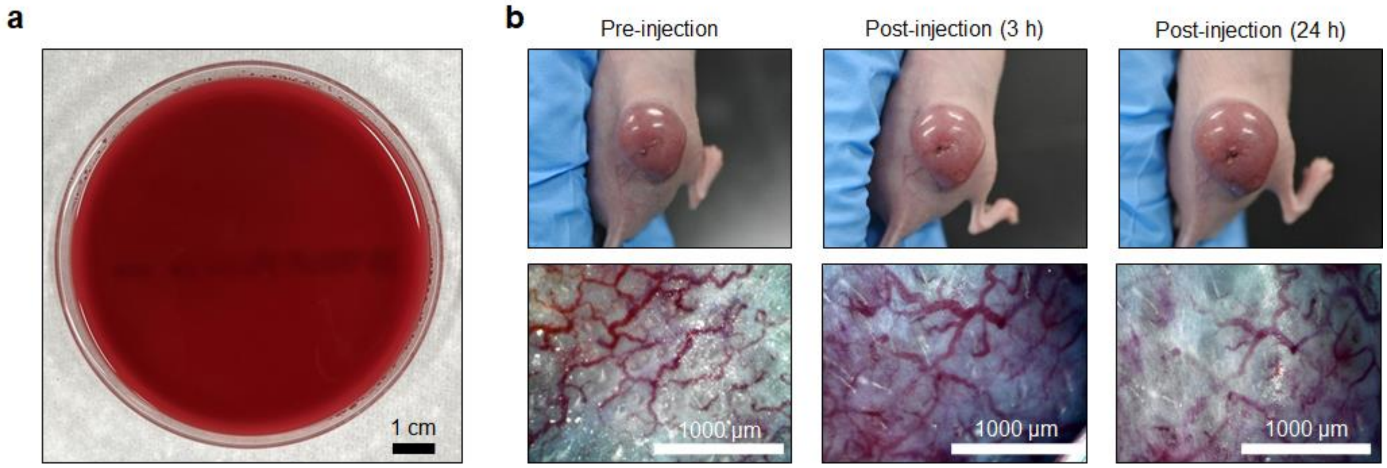

Supplementary Figure S40. (a) Photo of the control blood agar plate without bacterial inoculation. (b) Real-time observation of intratumoural blood vessels in Colon26 tumour–bearing BALB/c nude mice after i.v. injection of PBS (200 μL).

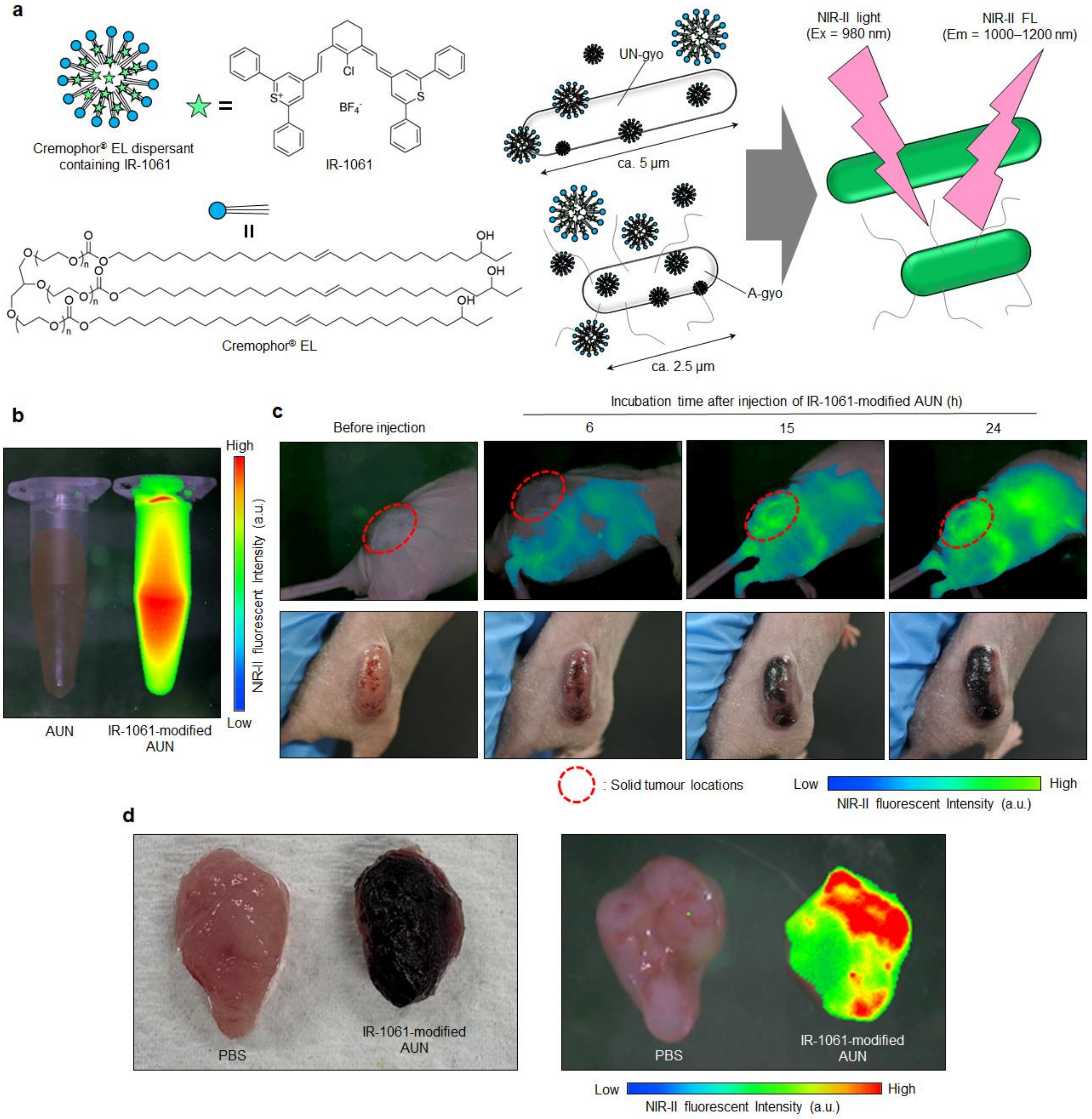

Supplementary Figure S41. Intratumour distribution of AUN. (a) Schematic illustration of IR-1061-modifed AUN. (b) NIR-II fluorescent images of AUN (left) and IR-1061-modifed AUN dispersions (right). (c) NIR-II fluorescent imaging of Colon26 tumour-bearing BALB/c nude mice after i.v. injection of IR-1061-modifed AUN. The images were taken 6 h, 15 h, and 24 h after i.v. injection of saline containing IR-1061-modifed AUN (200 µL, 2×10^9 CFU/mL). The control image also was shot before bacterial administration. The digital photos also were captured over time to observe the tumour discolouration caused by the bacterial destruction of intratumour vascular networks. The apparent tumour discolouration was observed around at 15 h after bacterial treatment. (d) Digital photo (left) and NIR-II fluorescent image (right) of the extracted tumours 24 h after an i.v. injection of PBS (200 µl) or IR-1061-modifed AUN (200 µL, 2×10^9 CFU/mL). The AUN treated tumour obviously showed discolouration due to the bacteria-mediated vascular destruction.

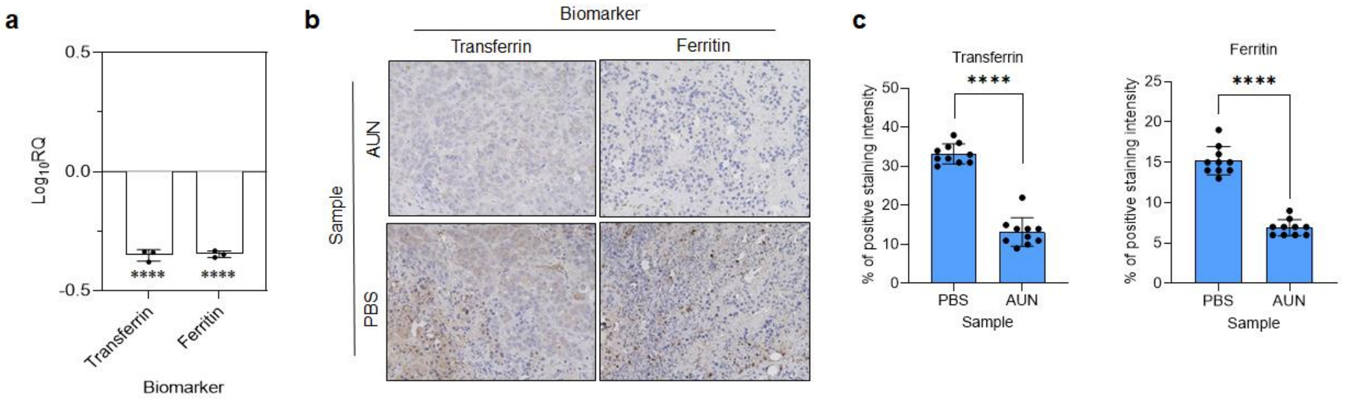

Supplementary Figure S42. Iron metabolism in the tumour microenvironment. (a) Quantitative polymerase chain reaction (qPCR) analyses of intratumour transferrin and ferritin. Quantification by qPCR of intratumour transferrin and ferritin 24 h after intravenous administration with AUN (200 µL, 5 × 10^9 CFU/mL). The mRNA expression of transferrin and ferritin are shown as fold change at log 10 relative quantification (RQ) relative to the control group (non-treatment). GAPDH gene expression was used as an internal control. Statistical significance was calculated in comparison with the non-treatment group. ****, $p < 0.0001$, by Student's two-sided *t*-test. Data are represented as mean ± standard errors of the mean (SEM); n = 3 independent tumour samples. (b) IHC staining of intratumour transferrin and ferritin. Tumour tissues were collected from Colon26 tumour-bearing BALB/c-nu/nu mice on day 1 after a single dose administration with AUN and PBS and stained for transferrin and ferritin. (c) Statistical analyses of cells positive for transferrin and ferritin positive from IHC staining. Data are represented as mean ± SEM; n = 10 independent areas (region of interest) in each tumour tissue collected from the groups of mice on day 1 after a single dose administration with AUN or PBS. Statistical significance was calculated in comparison with the PBS group. ****, $p < 0.0001$, by Student's two-sided *t*-test.

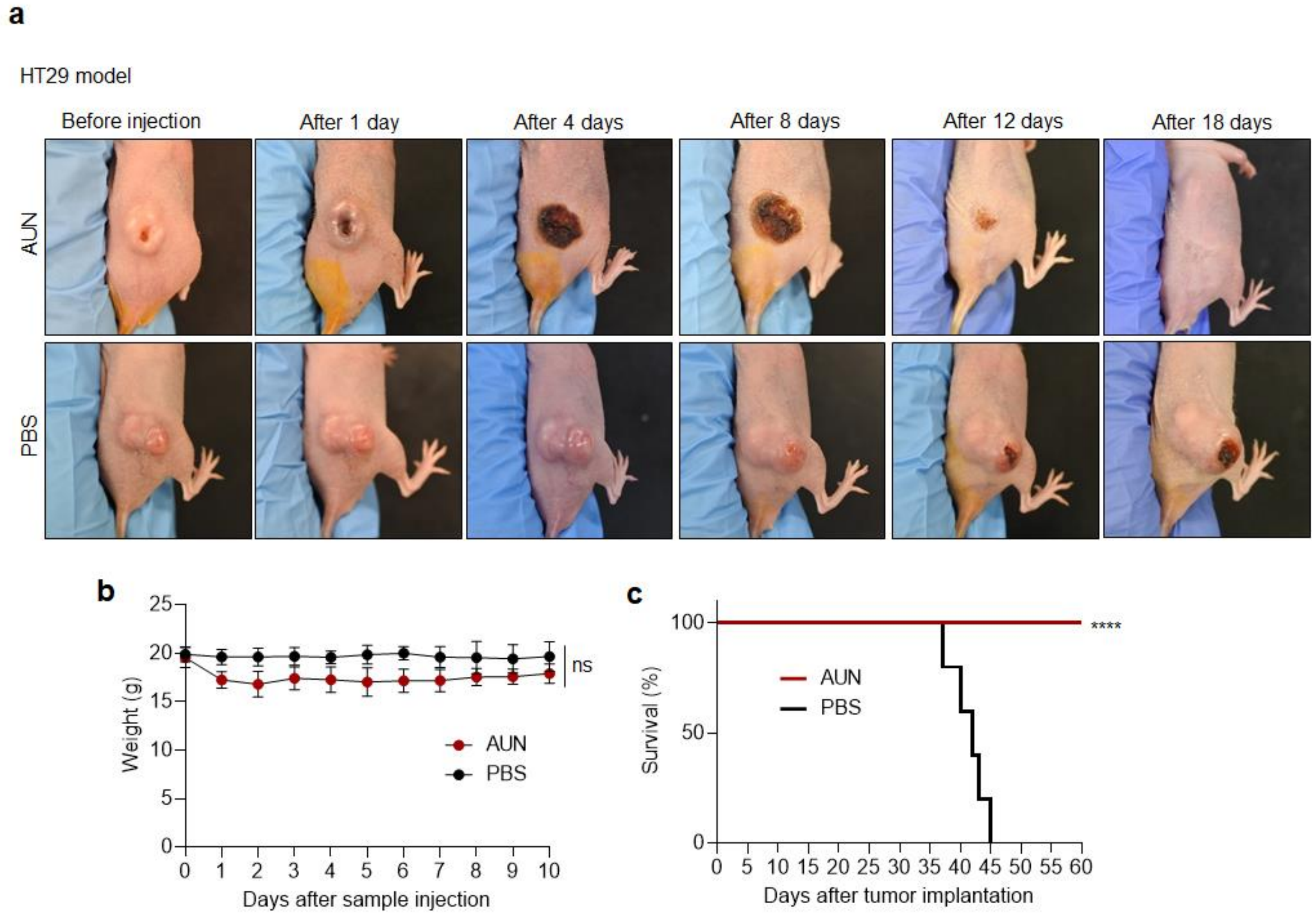

Supplementary Figure S43. *In vivo* antitumour efficacy of a double dose of AUN (1st dose, 1×10^7 CFU/mL; 2nd dose, 15×10^9 CFU/mL) against HT29 cancer model. (a) Images of mice after treatment with AUN or PBS. (b) Body weight measured after treatment with a double dose of AUN in HT29 tumour–bearing BALB/c nude mice. Statistical significance was calculated by comparison with the PBS group. Data are represented as mean ± standard errors of the mean (SEM); n = 5 independent experiments. ns, not significant ($p = 0.4223$), by two-way ANOVA. (c) Kaplan–Meier survival curves of Colon26 tumour–bearing BALB/c nude mice (n = 5 biologically independent mice) 60 days after tumour implantation. Statistical significance at the endpoint was calculated by comparison with the PBS group. ****, $p < 0.0001$, by log-rank (Mantel-Cox) test. Data are represented as mean ± SEM; n = 5 independent experiments.

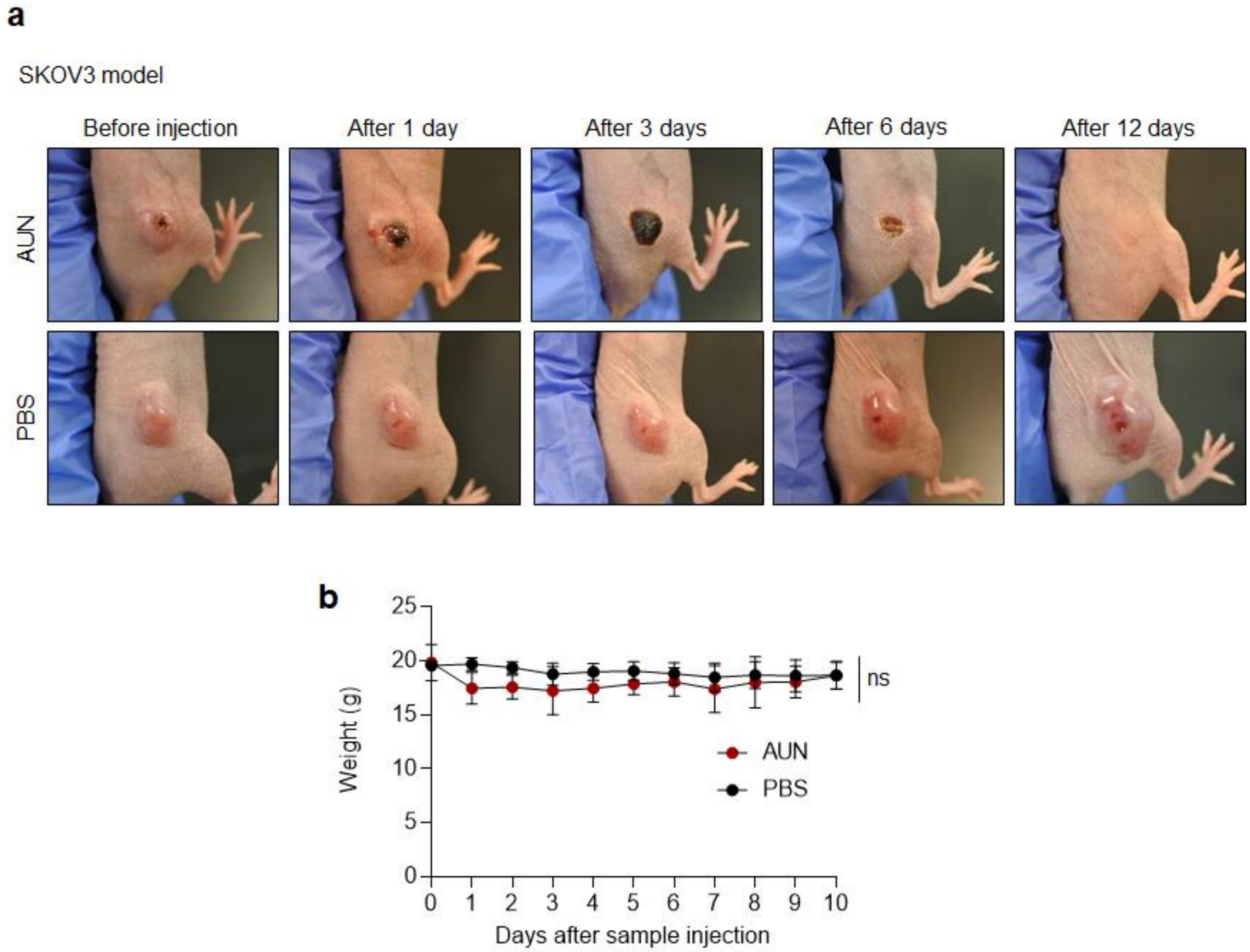

Supplementary Figure S44. *In vivo* antitumour efficacy of a double dose of AUN (1st dose, 1×10^7 CFU/mL; 2nd dose, 15×10^9 CFU/mL) against SKOV3 cancer model. (a) Images of mice after treatment with AUN or PBS. (b) Body weight measured after treatment with a double dose of AUN in SKOV3 tumour–bearing BALB/c nude mice. Statistical significance was calculated by comparison with the PBS group. Data are represented as mean ± standard errors of the mean; n = 5 independent experiments. ns, not significant (p = 0.6487), by two-way ANOVA test.

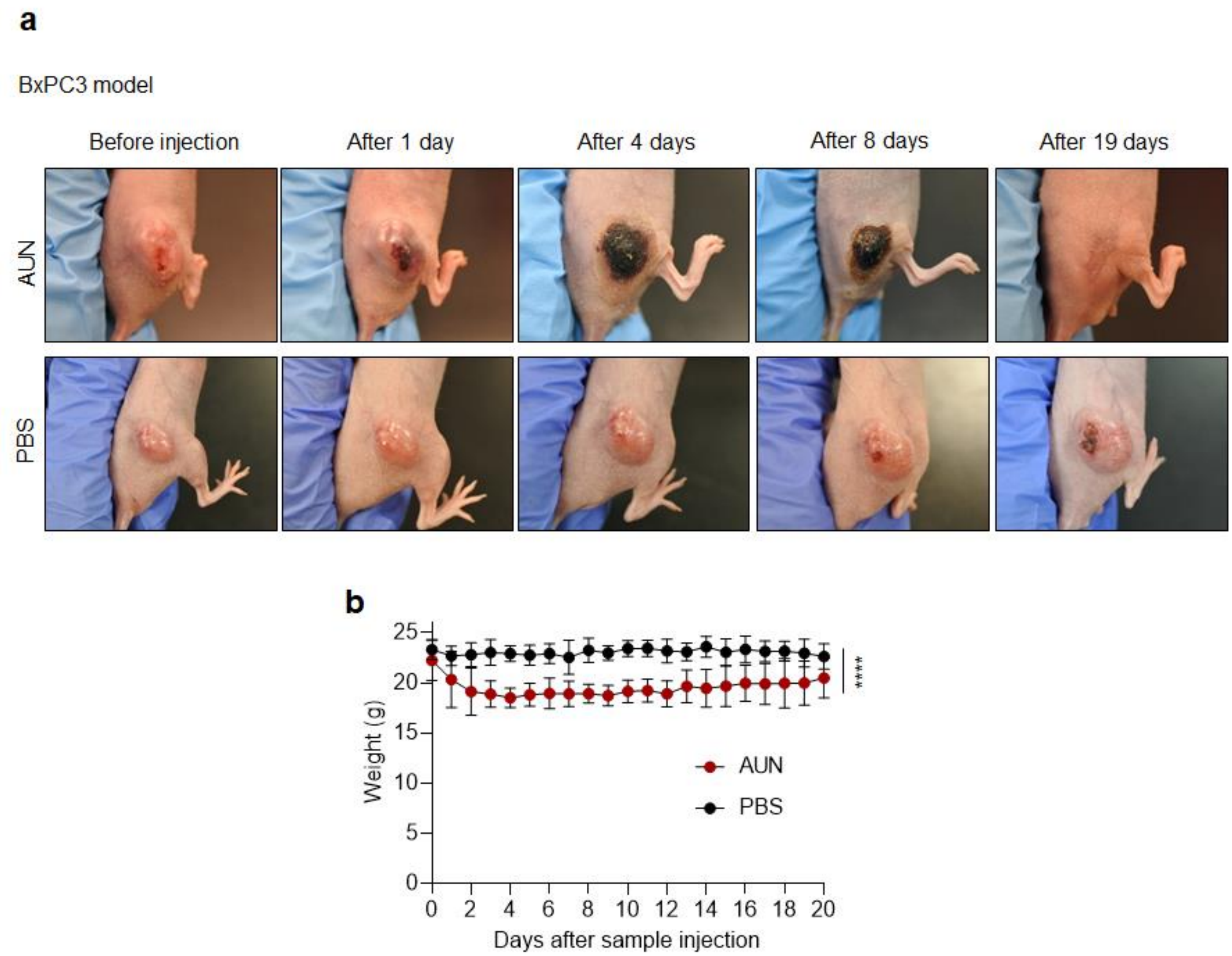

Supplementary Figure S45. *In vivo* antitumour efficacy of a double dose of AUN (1st dose, 1×10^9 CFU/mL; 2nd dose, 20×10^9 CFU/mL) against BxPC3 cancer model. (a) Images of mice after treatment with AUN or PBS. (b) Body weight measured after treatment with a double dose of AUN in BxPC3 tumour–bearing BALB/c nude mice. Statistical significance was calculated by comparison with the PBS group. Data are represented as mean ± standard errors of the mean; n = 5 independent experiments. ****, $p < 0.0001$, by two-way ANOVA test.

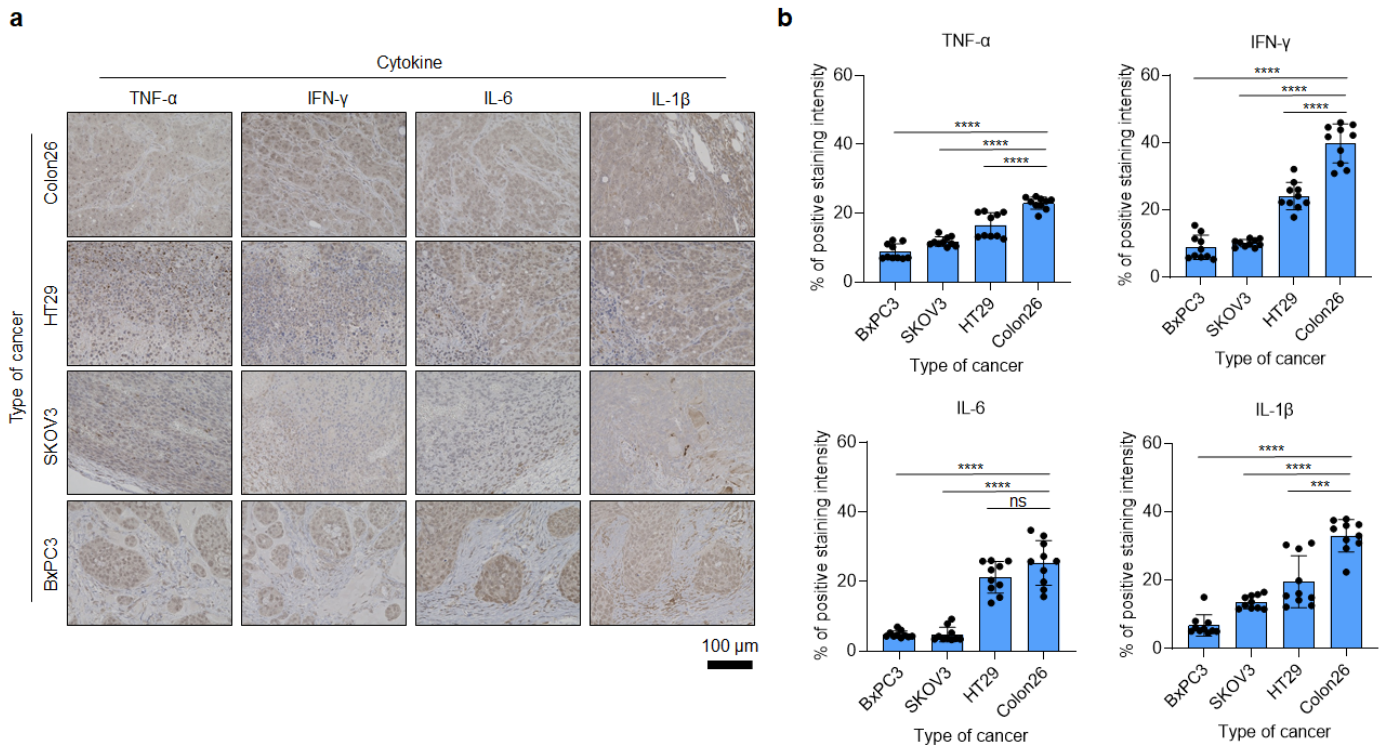

Supplementary Figure S46. Inflammatory cytokine expression behaviours in the various tumours of BALB/c-nu/nu mice after treatment with AUN, measured by the IHC staining method. (a) Tumour tissues were collected from Colon26, HT29, SKOV3, or BxPC3 tumour-bearing BALB/c-nu/nu mice on day 1 after a single dose administration with AUN (200 μL, 5×10^9 CFU/mL) and stained for TNF-α, IFN-γ, IL-6, and IL-1β. (b) Statistical analyses of cells positive for TNF-α, IFN-γ, IL-6, and IL-1β positive. Data are represented as mean ± standard error of the mean; n = 10 independent areas (region of interest) in each tumour tissue collected from the groups of mice on day 1 after a single dose administration with AUN. Statistical significance was assessed using a two-sided Student's t-test for comparisons between Colon26 and HT29, and one-way ANOVA for comparisons among three or more tumour types. ns, not significant (p = 0.1109); ***, p = 0.0002; ****, p < 0.0001.

Supplemental Table S1. Genes and products against each gene IDs in **Figure 1f**.

Gene ID	Gene	Product
UN-gyo_00226	*egtD*	Histidine N-alpha-methyltransferase
UN-gyo_00651		Putative cytokinin riboside 5'-monophosphate phosphoribohydrolase
UN-gyo_00778		hypothetical protein
UN-gyo_00779	*isiB*	Flavodoxin
UN-gyo_00780	*hmuV*	Hemin import ATP-binding protein HmuV
UN-gyo_00781	*hmuU_1*	Hemin transport system permease protein HmuU
UN-gyo_00782	*btuF*	Vitamin B12-binding protein
UN-gyo_00783		hypothetical protein
UN-gyo_00784	*chuW*	Anaerobilin synthase
UN-gyo_00785		hypothetical protein
UN-gyo_00786	*hxuC*	Heme/hemopexin utilization protein C
UN-gyo_00787	*isdG*	Heme oxygenase (staphylobilin-producing)
UN-gyo_00788	*hutX*	Intracellular heme transport protein HutX
UN-gyo_00789	*exbB_1*	Biopolymer transport protein ExbB
UN-gyo_00790	*exbD_1*	Biopolymer transport protein ExbD
UN-gyo_00791		hypothetical protein
UN-gyo_00792		hypothetical protein
UN-gyo_00853		hypothetical protein
UN-gyo_00854		hypothetical protein
UN-gyo_00859	*cutL_2*	Carbon monoxide dehydrogenase large chain
UN-gyo_00883		hypothetical protein
UN-gyo_00919	*yceJ_1*	Cytochrome b561
UN-gyo_00924		hypothetical protein
UN-gyo_00925		hypothetical protein
UN-gyo_00926		hypothetical protein
UN-gyo_01135		Solute-binding protein
UN-gyo_01788		hypothetical protein
UN-gyo_01814		putative transporter
UN-gyo_01877	*qheDH*	Quinohemoprotein alcohol dehydrogenase
UN-gyo_01906		hypothetical protein
UN-gyo_02164	*ycjP*	Inner membrane ABC transporter permease protein YcjP
UN-gyo_02166		hypothetical protein
UN-gyo_02209	*yadG*	putative ABC transporter ATP-binding protein YadG
UN-gyo_02293		hypothetical protein
UN-gyo_02381	*infA*	Translation initiation factor IF-1
UN-gyo_02643		hypothetical protein
UN-gyo_02763		hypothetical protein
UN-gyo_02884		hypothetical protein

UN-gyo_03084	*ttgB_2*	Toluene efflux pump membrane transporter TtgB
UN-gyo_03085	*mdtE_2*	Multidrug resistance protein MdtE
UN-gyo_03108	*vgb*	Virginiamycin B lyase
UN-gyo_03111		hypothetical protein
UN-gyo_03361	*cutL_4*	Carbon monoxide dehydrogenase large chain
UN-gyo_03362	*cutM*	Carbon monoxide dehydrogenase medium chain
UN-gyo_03377		hypothetical protein
UN-gyo_03765		hypothetical protein
UN-gyo_04122		hypothetical protein
UN-gyo_04137		hypothetical protein
UN-gyo_04227	*hbaA*	4-hydroxybenzoate--CoA/benzoate--CoA ligase
UN-gyo_04228	*hcrC*	4-hydroxybenzoyl-CoA reductase subunit gamma
UN-gyo_04305		Long-chain-fatty-acid--CoA ligase FadD13
UN-gyo_04306	*iro*	Iron oxidase
UN-gyo_04307		hypothetical protein
UN-gyo_04326		hypothetical protein
UN-gyo_04327	*btuB_7*	Vitamin B12 transporter BtuB
UN-gyo_04452	*ligXa*	5,5'-dehydrodivanillate O-demethylase oxygenase subunit
UN-gyo_04587		hypothetical protein
UN-gyo_04797		hypothetical protein

Supplemental Table S2. Viability of AUN-injected mice after administration of various immunostimulants or anticoagulation heparin.

Sample		Administration way	Dose (μg/head)	Survival rate (%)******
AUN alone		i.v.	*	0
Neutrophil depletion**	Anti-Ly6G antibody	i.p.	2×10^2	100
Immunostimulant***	Poly (I:C)	i.v.	200	0
	GM-CSF	i.v.	1	0
	Vitamin D_3	i.v.	200	0
	Imiquimod	i.p.	400	0
Anticoagulation****	Heparin	i.v.	5×10^3	100
Anti-inflammatory agent*****	Dexamethasone	s.c.	2×10^3	100

*AUN (200 μL, 15×10^9 CFU/mL) was i.v. injected to BALB/c-nu/nu mice (n = 5). Mice died within one or two days after i.v. administration when given more than 7.8×10^9 CFU/mL of AUN (200 μL).

***AUN (200 μL, 15×10^9 CFU/mL) was i.v. injected to BALB/c-nu/nu mice (n = 5) 24 h after administration of Anti-Ly6G antibody.

***AUN (200 μL, 15×10^9 CFU/mL) was i.v. injected to BALB/c-nu/nu mice (n = 5) 48 h after administration of each immunostimulant.

****AUN (200 μL, 15×10^9 CFU/mL) was i.v. injected to BALB/c-nu/nu mice (n = 5) 1 h after administration of heparin.

*****AUN (200 μL, 15×10^9 CFU/mL) was i.v. injected to BALB/c-nu/nu mice (n = 5) 48 h after administration of dexamethasone.

******Survival rate was measured once after bacterial injection for 30 days.

Table S3. Biochemical parameters of the BALB/c-nu/nu mice after double-shot of AUN (1×10^7 CFU/mL and 15×10^9 CFU/mL) for 14 days.

Measured value	Entry	Unit	PBS (placebo) (n = 5)	Double-shot of AUN (n = 5)	P value	Remarks
Biochemical parameters	TP	g dL^{-1}	4.9 ± 0.10	5.0 ± 0.15	ns (0.1079)	Within normal limits
	ALB	g dL^{-1}	3.1 ± 0.08	2.9 ± 0.04	0.0111	Within normal limits
	BUN	mg dL^{-1}	23.8 ± 0.90	24.0 ± 1.90	ns (0.8819)	Within normal limits
	CRE	mg dL^{-1}	0.1 ± 0.01	0.1 ± 0.01	ns (0.8149)	Within normal limits
	Na	mEq L^{-1}	145.0 ± 1.73	145.8 ± 0.84	ns (0.3375)	Within normal limits
	K	mEq L^{-1}	22.9 ± 0.73	22.7 ± 1.52	ns (0.7993)	Within normal limits
	Cl	mEq L^{-1}	106.4 ± 0.55	105.8 ± 0.84	ns (0.3046)	Within normal limits
	AST	IU L^{-1}	43.8 ± 0.84	64.2 ± 6.34	0.0017	Within normal limits
	ALT	IU L^{-1}	20.2 ± 0.84	28.4 ± 2.51	0.0038	Within normal limits
	LDH	IU L^{-1}	161.8 ± 9.76	202.2 ± 18.10	0.0013	Within normal limits
	AMY	IU L^{-1}	2050.0 ± 203.80	2101.2 ± 140.71	ns (0.7375)	Within normal limits
	CK	IU L^{-1}	56.6 ± 4.88	57.8 ± 4.09	ns (0.5685)	Within normal limits

Data are represented as the mean ± standard errors of the mean; n = 5 biologically independent mice. Statistical analyses were performed using the Student's two-sided *t*-test. ns, not significant. We referred the normal limits of biochemical parameters in the manufacture's technical information.

Abbreviations: ALB, albumin; ALT, alanine transaminase; AMY, amylase; AST, aspartate aminotransferase; BUN, blood urea nitrogen; Cl, chlorine; CK, creatine kinase; CRE, creatinine; K, potassium; LDH, lactate dehydrogenase; Na, sodium; TP, total protein.

Table S4. Biochemical parameters of the SCID mice after double-shot of AUN (1×10^7 CFU/mL and 7×10^9 CFU/mL) for 14 days.

Measured value	Entry	Unit	PBS (placebo) (n = 5)	Double-shot of AUN (n = 5)	P value	Remarks
Biochemical parameters	TP	g dL^{-1}	4.8 ± 0.11	4.8 ± 0.08	ns (0.6213)	Within normal limits
	ALB	g dL^{-1}	3.1 ± 0.07	3.2 ± 0.10	ns (0.0890)	Within normal limits
	BUN	mg dL^{-1}	16.5 ± 2.84	22.3 ± 1.35	0.0188	Within normal limits
	CRE	mg dL^{-1}	0.1 ± 0.02	0.1 ± 0.03	ns (> 0.9999)	Within normal limits
	Na	mEq L^{-1}	142.8 ± 1.48	143.0 ± 0.71	ns (0.8149)	Within normal limits
	K	mEq L^{-1}	24.6 ± 0.83	24.1 ± 1.41	ns (0.3292)	Within normal limits
	Cl	mEq L^{-1}	102.2 ± 1.30	103.0 ± 0.71	ns (0.242)	Within normal limits
	AST	IU L^{-1}	39.2 ± 2.68	43.6 ± 0.55	0.0109	Within normal limits
	ALT	IU L^{-1}	21.8 ± 2.59	18.4 ± 2.07	ns (0.0817)	Within normal limits
	LDH	IU L^{-1}	152.0 ± 17.15	195.2 ± 44.97	ns (0.0774)	Within normal limits
	AMY	IU L^{-1}	2018.8 ± 209.36	2254.2 ± 196.70	ns (0.1472)	Within normal limits
	CK	IU L^{-1}	60.0 ± 7.00	73.8 ± 16.24	ns (0.15)	Within normal limits

Data are represented as the mean ±standard errors of the mean; n = 5 biologically independent mice. Statistical analyses were performed using the Student's two-sided *t*-test. ns, not significant,. We referred the normal limits of biochemical parameters in the manufacture's technical information.

Abbreviations: ALB, albumin; ALT, alanine transaminase; AMY, amylase; AST, aspartate aminotransferase; BUN, blood urea nitrogen; Cl, chlorine; CK, creatine kinase; CRE, creatinine; K, potassium; LDH, lactate dehydrogenase; Na, sodium; TP, total protein.

Table S5. Biochemical parameters of the NOD-SCID mice after double-shot of AUN (1×10^8 CFU/mL and 15×10^9 CFU/mL) for 14 days.

Measured value	Entry	Unit	PBS (placebo) (n = 5)	Double-shot of AUN (n = 5)	P value	Remarks
Biochemical parameters	TP	g dL^{-1}	4.5 ± 0.13	5.3 ± 0.08	0.0001	Within normal limits
	ALB	g dL^{-1}	3.0 ± 0.19	3.1 ± 0.12	ns (0.1836)	Within normal limits
	BUN	mg dL^{-1}	30.8 ± 1.97	34.1 ± 1.29	ns (0.8819)	Within normal limits
	CRE	mg dL^{-1}	0.1 ± 0.01	0.1 ± 0.01	ns (> 0.9999)	Within normal limits
	Na	mEq L^{-1}	144.6 ± 2.19	147.4 ± 1.14	ns (0.3375)	Within normal limits
	K	mEq L^{-1}	20.4 ± 0.65	21.9 ± 0.7	ns (0.7993)	Within normal limits
	Cl	mEq L^{-1}	102.6 ± 1.34	104.4 ± 1.14	ns (0.3046)	Within normal limits
	AST	IU L^{-1}	49.0 ± 8.34	129.6 ± 26.29	0.004	Within normal limits
	ALT	IU L^{-1}	20.0 ± 4.80	96.0 ± 56.25	0.0367	Within normal limits
	LDH	IU L^{-1}	185.4 ± 20.82	255.6 ± 37.65	0.0088	Within normal limits
	AMY	IU L^{-1}	2036.2 ± 297.00	2742.6 ± 331.42	0.0335	Within normal limits
	CK	IU L^{-1}	94.6 ± 39.26	81.6 ± 30.03	ns (0.5685)	Within normal limits

Data are represented as the mean ± standard errors of the mean; n = 5 biologically independent mice. Statistical analyses were performed using the Student's two-sided *t*-test. ns, not significant. We referred the normal limits of biochemical parameters in the manufacture's technical information.

Abbreviations: ALB, albumin; ALT, alanine transaminase; AMY, amylase; AST, aspartate aminotransferase; BUN, blood urea nitrogen; Cl, chlorine; CK, creatine kinase; CRE, creatinine; K, potassium; LDH, lactate dehydrogenase; Na, sodium; TP, total protein.

Supplementary Table S6. Complete blood counts (CBCs) and biochemical parameters of the lower-limb ischemia model mice injected with PBS (200 μL) or AUN (200 μL, 5×10^9 CFU/mL) dispersion after 30 days.

Measured value	Entry	Unit	PBS (placebo) (n = 5)	AUN (n = 5)	P value
CBC	WBC	×10^2 /μL	51.80 ± 4.84	46.60 ± 2.84	ns (0.3813)
	RBC	×10^4 /μL	800.70 ± 3.28	814.00 ± 4.36	ns (0.0709)
	HGB	g/dL	13.92 ± 0.11	14.14 ± 0.13	ns (0.2397)
	HCT	%	41.25 ± 2.4	43.00 ± 0.42	ns (0.06)
	MCV	fL	51.56 ± 0.24	51.62 ± 0.37	ns (0.8958)
	MCH	pg	17.46 ± 0.10	17.12 ± 0.11	ns (0.0511)
	MCHC	g/dL	33.77 ± 0.18	33.53 ± 0.12	ns (0.3357)
	PLT	×10^4 /μL	81.44 ± 2.60	87.32 ± 3.51	ns (0.2151)
Biochemical parameters	TP	g/dL	4.40 ± 0.09	4.54 ± 0.02	ns (0.1909)
	ALB	g/dL	3.04 ± 0.05	3.02 ± 0.02	ns (0.7245)
	BUN	mg/dL	24.02 ± 1.34	24.00 ± 1.36	ns (0.9919)
	CRE	mg/dL	0.13 ± 0.01	0.13 ± 0.01	ns (0.6406)
	Na	mEq/L	146.40 ± 0.81	147.00 ± 0.63	ns (0.5761)
	K	mEq/L	21.15 ± 0.45	20.35 ± 0.05	ns (0.1109)
	Cl	mEq/L	107.40 ± 0.87	108.40 ± 0.60	ns (0.3724)
	AST	IU/L	39.60 ± 1.29	40.40 ± 1.03	ns (0.6406)
	ALT	IU/L	19.60 ± 1.12	19.60 ± 1.29	ns (> 0.9999)
	LDH	IU/L	153.00 ± 4.25	150.60 ± 4.25	ns (0.7003)
	AMY	IU/L	2755.40 ± 119.48	2766.60 ± 50.65	ns (0.9333)
	CK	IU/L	44.2 ± 2.11	45.80 ± 1.80	ns (0.5796)

Data are represented as means ± standard errors of the mean; n = 5 biologically independent mice. Statistical analyses comprise the Student's two-sided *t*-test. ns, not significant.

Abbreviations: ALB, albumin; ALT, alanine transaminase; AMY, amylase; AST, aspartate aminotransferase; BUN, blood urea nitrogen; Cl, chlorine; CK, creatine kinase; CRE, creatinine; HCT, haematocrit; HGB, haemoglobin; K, potassium; LDH, lactate dehydrogenase; MCH, mean corpuscular haemoglobin; MCHC, mean corpuscular haemoglobin concentration; MCV, mean corpuscular volume; Na, sodium; PLT, platelet; RBC, red blood cell; TP, total protein; WBC, white blood cell.

Supplemental Table S7. Antibodies used in this study.

Antibody	Type	Source	Catalogue No.	Application
F4/80	Mouse Monoclonal	BMA Biomedicals	T-2028	IHC (1:50)
CD3	Rabbit Monoclonal	Abcam	ab16669	IHC (1:100)
CD19	Rabbit Polyclonal	Bioss	bs-0079R	IHC (1:100)
CXCR4	Goat Polyclonal	Abcam	ab1670	IHC (1:100)
NKp46	Rabbit Polyclonal	Affinity Biosciences	DF7599	IHC (1:100)
Caspase-3	Rabbit Polyclonal	Cell Signaling Technology	9661S	IHC (1:100)
TNF-α	Rabbit Polyclonal	Abcam	ab6671	IHC (1:100)
IFN-γ	Rabbit Polyclonal	Abcam	Ab9657	IHC (1:100)
IL-6	Rabbit Polyclonal	Bioss	bs-0782R	IHC (1:100)
IL-1β	Rabbit Polyclonal	GenTex	GTX100793	IHC (1:100)
Fibrin	Mouse Monoclonal	Merck	MABS2155-25UG	IHC (1:50)
Transferrin	Rabbit Polyclonal	Proteintech	17435-1-AP	IHC (1:100)
Ferritin	Rabbit Polyclonal	Proteintech	11682-1-AP	IHC (1:100)
CA19-9	Mouse Monoclonal	Abcam	ab289665	IHC (1:500)
Digoxigenin-peroxidase	Sheep Polyclonal	Merck Millipore	S7100	IHC (Tunel) (Non-dilution)
FITC–CD3	Human cell line Monoclonal	Miltenyi Biotech	130-119-798	Flow cytometry
PE–CD335 (NKp46)	Human cell line Monoclonal	Miltenyi Biotech	130-112-358	Flow cytometry
PerCP-Vio 700–CD45R (B220)	Human cell line Monoclonal	Miltenyi Biotech	130-110-850	Flow cytometry
PE-Vio 770–Ly-6G	Human cell line	Miltenyi Biotech	130-121-438	Flow cytometry

	Monoclonal			
APC–F4/80	Human cell line Monoclonal	Miltenyi Biotech	130-116-525	Flow cytometry
APC-Vio 770–CD45	Human cell line Monoclonal	Miltenyi Biotech	130-110-800	Flow cytometry

Supplemental Table S8. TaqMan™ Primers for qPCR.

Target	Assay ID	Gene Symbol	Source
F4/80	Mm00802529_m1	Adgre1	Thermo Fisher Scientific
CD3	Mm00442746_m1	Cd3d	Thermo Fisher Scientific
CD19	Mm00515420_m1	Cd19	Thermo Fisher Scientific
CXCR4	Mm01996749_s1	Cxcr4	Thermo Fisher Scientific
NKp46	Mm01337324_g1	Ncr1	Thermo Fisher Scientific
IFN-γ	Mm01168134_m1	Ifng	Thermo Fisher Scientific
TNF-α	Mm00443258_m1	Tnf	Thermo Fisher Scientific
GAPDH	Mm99999915_g1	Gapdh	Thermo Fisher Scientific

Table S9. 16s rDNA primers used for RT-qPCR.

Bacteria	Forward (5'-3')	Reverse (5'-3')	Length (bp)
UN-gyo (*R. palustris*)	GCGGGAAGATAATGACGGTACC	CAGGCTTTCACCTCTGACTTAG	147
A-gyo (*P. mirabilis*)	GGAGGGTGCAAGCGTTAATCGG	CTACAAGACTCTAGCCAACCAG	128
