# Supplementary material for: Tumour-resident oncolytic bacteria trigger potent anticancer effects through selective intratumoural thrombosis and necrosis
Source: Nat Biomed Eng. 2025 Aug 5;10(2):293–308. doi: 10.1038/s41551-025-01459-9 (PMC12920090; doi:10.1038/s41551-025-01459-9)
Supplement: Supplementary file 2 — Reporting Summary [file 41551_2025_1459_MOESM2_ESM.pdf]

nature portfolio

Corresponding author(s): Eijiro Miyako

Last updated by author(s): Jun 6, 2025

# Reporting Summary

Nature Portfolio wishes to improve the reproducibility of the work that we publish. This form provides structure for consistency and transparency in reporting. For further information on Nature Portfolio policies, see our Editorial Policies and the Editorial Policy Checklist.

## Statistics

For all statistical analyses, confirm that the following items are present in the figure legend, table legend, main text, or Methods section.

| n/a | Confirmed | |
|---|---|---|
| ☐ | ☒ | The exact sample size (*n*) for each experimental group/condition, given as a discrete number and unit of measurement |
| ☐ | ☒ | A statement on whether measurements were taken from distinct samples or whether the same sample was measured repeatedly |
| ☐ | ☒ | The statistical test(s) used AND whether they are one- or two-sided<br>*Only common tests should be described solely by name; describe more complex techniques in the Methods section.* |
| ☒ | ☐ | A description of all covariates tested |
| ☒ | ☐ | A description of any assumptions or corrections, such as tests of normality and adjustment for multiple comparisons |
| ☐ | ☒ | A full description of the statistical parameters including central tendency (e.g. means) or other basic estimates (e.g. regression coefficient) AND variation (e.g. standard deviation) or associated estimates of uncertainty (e.g. confidence intervals) |
| ☐ | ☒ | For null hypothesis testing, the test statistic (e.g. *F*, *t*, *r*) with confidence intervals, effect sizes, degrees of freedom and *P* value noted<br>*Give P values as exact values whenever suitable.* |
| ☒ | ☐ | For Bayesian analysis, information on the choice of priors and Markov chain Monte Carlo settings |
| ☒ | ☐ | For hierarchical and complex designs, identification of the appropriate level for tests and full reporting of outcomes |
| ☒ | ☐ | Estimates of effect sizes (e.g. Cohen's *d*, Pearson's *r*), indicating how they were calculated |

*Our web collection on statistics for biologists contains articles on many of the points above.*

## Software and code

Policy information about availability of computer code

| | |
|---|---|
| Data collection | BZ-X800 Analyzer V1.1.2.4; CellSens V3.1; GOKO Measure Plus V2.0.0; MACSQuantify V2.13.3 |
| Data analysis | Excel 2016; GraphPad Prism 9.4.0; MACSQuantify V2.13.3; BZ-X800 Analyzer Ver1.1.2.4 |

For manuscripts utilizing custom algorithms or software that are central to the research but not yet described in published literature, software must be made available to editors and reviewers. We strongly encourage code deposition in a community repository (e.g. GitHub). See the Nature Portfolio guidelines for submitting code & software for further information.

## Data

Policy information about availability of data

All manuscripts must include a data availability statement. This statement should provide the following information, where applicable:

- Accession codes, unique identifiers, or web links for publicly available datasets
- A description of any restrictions on data availability
- For clinical datasets or third party data, please ensure that the statement adheres to our policy

The main data supporting the results in this study are available within the paper and its Supplementary Information. The raw and analysed datasets from the study are too large to be publicly shared, but they are available for research purposes from the corresponding author on reasonable request. Source data are provided with this paper.

## Research involving human participants, their data, or biological material

Policy information about studies with human participants or human data. See also policy information about sex, gender (identity/presentation), and sexual orientation and race, ethnicity and racism.

| | |
|---|---|
| Reporting on sex and gender | The donor was male. Sex was not a variable in the study design or analysis. |
| Reporting on race, ethnicity, or other socially relevant groupings | The donor was reported by the supplier as Black and Non-Hispanic. Race and ethnicity were not variables in the study design or analysis. |
| Population characteristics | The study used commercially available refrigerated human blood obtained from a single healthy donor, aged 32 years, identified as male, Black, and Non-Hispanic. |
| Recruitment | No direct recruitment was performed. The blood sample was purchased from a commercial supplier [BioIVT (College Park, MD, USA)], and donor information was limited to age, sex, and self-reported race/ethnicity. |
| Ethics oversight | Ethical approval was not required as the study used anonymized, commercially obtained human biological material with no identifiable personal information. |

Note that full information on the approval of the study protocol must also be provided in the manuscript.

# Field-specific reporting

Please select the one below that is the best fit for your research. If you are not sure, read the appropriate sections before making your selection.

☒ Life sciences ☐ Behavioural & social sciences ☐ Ecological, evolutionary & environmental sciences

For a reference copy of the document with all sections, see nature.com/documents/nr-reporting-summary-flat.pdf

# Life sciences study design

All studies must disclose on these points even when the disclosure is negative.

| | |
|---|---|
| Sample size | For experiments involving cell-free experiments, in vitro cell experiments, and phenotype analysis of the cells in tissues, n = 3 was chosen as the minimal replicate numbers. For in vivo antitumour experiments, n = 5 was chosen as the minimal replicate numbers. Sample sizes in the animal studies were determined on the basis of previous experimental experience (Xi Yang et al. Nano Today 37, 101100 (2021); Sheethal Reghu et al. Nano Today 52, 101966 (2023); Yun Qi et al. Advanced Functional Materials 34, 2305886 (2023). ) |
| Data exclusions | No data was excluded from this study |
| Replication | Experiments were repeated at least three independent experiments with similar results. All experiments were reproduced to reliably support conclusions stated in the manuscript. |
| Randomization | For the in vivo studies, the animals were randomly assigned to treatment groups at the outset of the study. Mice were assigned consecutive numbers across cages, and the groups were clustered consecutive numbers at random. All animals were sex- and age-matched within each experimental group. Different experiments used animals of different ages as appropriate. Male mice were used only in the BxPC3 orthotopic pancreatic tumour model; all other experiments employed female mice. No animals were excluded based on sex or age, and no selection bias was introduced in assigning animals to treatment groups. |
| Blinding | Investigators were blinded to group allocation during the experiments. |

# Reporting for specific materials, systems and methods

We require information from authors about some types of materials, experimental systems and methods used in many studies. Here, indicate whether each material, system or method listed is relevant to your study. If you are not sure if a list item applies to your research, read the appropriate section before selecting a response.

## Materials & experimental systems

| n/a | Involved in the study | |
|---|---|---|
| ☐ | ☒ | Antibodies |
| ☐ | ☒ | Eukaryotic cell lines |
| ☒ | ☐ | Palaeontology and archaeology |
| ☐ | ☒ | Animals and other organisms |
| ☒ | ☐ | Clinical data |
| ☒ | ☐ | Dual use research of concern |
| ☒ | ☐ | Plants |

## Methods

| n/a | Involved in the study | |
|---|---|---|
| ☒ | ☐ | ChIP-seq |
| ☐ | ☒ | Flow cytometry |
| ☒ | ☐ | MRI-based neuroimaging |

# Antibodies

Antibodies used

Antibodies against F4/80 (mouse monoclonal, T-2028, BMA Biomedicals, 1:50), CD3 (rabbit monoclonal, ab16669, Abcam, 1:100), CD19 (rabit polyclonal, bs-0079R, Bioss, 1:100), CXCR4 (goat polyclonal, ab1670, Abcam, 1:100), Nkp46 (rabbit polyclonal, DF7599, Affinity Biosciences, 1:100),  Caspase-3 (Rabbit polyclonal, 9661S, Cell Signaling Technology, 1:00), TNF-α (Rabbit polyclonal, ab6671, Abcam, 1:100), IFN-γ (rabbit polyclonal, ab9657, Abcam, 1:100), IL-6 (rabbit polyclonal, bs-0782R, Bioss, 1:100), IL-1β (rabbit polyclonal, GTX100793, GenTex, 1:100), Fibrin (mouse monoclonal, MABS2155-25UG, Merck, 1:50), Transferrin (rabbit polyclonal, 17435-1-AP, Proteintech, 1:100), Ferritin (rabbit polyclonal, 11682-1-AP, Proteintech, 1:100), CA19-9 (mouse monoclonal, ab289665, Abcam, 1:500), and digoxigenin-peroxidase (sheep polyclonal, S7100, Merck Millipore, non-dilution) were used for IHC staining. Antibodies against CD3 (FITC-labelled, Human cell line monoclonal, 130119-798, Miltenyi Biotech), CD335 (NKp46) (PE-labelled, Human cell line monoclonal, 130-112-358, Miltenyi Biotech), CD45R (B220) (PerCP-Vio 700-labelled, Human cell line monoclonal, 130-110-850, Miltenyi Biotech), Ly-6G (PE-Vio 770-labelled, Human cell line monoclonal, 130-121-438, Miltenyi Biotech), F4/80 (APC-labelled, Human cell line monoclonal, 130-116-525, Miltenyi Biotech), and CD45 (APC-Vio 770-labelled, Human cell line monoclonal, 130-110-800, Miltenyi Biotech) were used for flow cytometry.

Validation

All antibodies were used in the study according to the profile of manufacturers. Antibody validation for preparation of immunohistochemistry or flowcytometry was validated by the supplier and confirmed in Figure 3 and Supplementary Figures 9, 26, 42, and 46.

# Eukaryotic cell lines

Policy information about cell lines and Sex and Gender in Research

Cell line source(s)

The Colon26 cell line (catalogue no. RBC2657) was obtained from RIKEN BioResource Research Center (Ibaraki, Japan). Human normal diploid fibroblasts (MRC5) cell line (catalogue no. JCRB9008) was obtained from the Japanese Collection of Research Bioresources Cell Bank (Tokyo, Japan). Human pancreatic cancer (BxPC3) cell (catalogue no. CRL1687) was obtained from American Type Culture Collection (Manassas, VA, USA). Human ovarian cancer (SKOV3) (catalogue no. EC91091004-F0) and human colorectal adenocarcinoma (HT29) cells (catalogue no. EC91072201-G0) cells were obtained from KAC Co., Ltd. (Tokyo, Japan).

Authentication

These cell lines were authenticated by the supplier using STR analysis.

Mycoplasma contamination

No contamination was detected by the supplier using Hoechst DNA stain method, agar culture method, PCR-based assay.

Commonly misidentified lines
(See ICLAC register)

These cell lines that we used were not listed in commonly misidentified lines in ICLAC Register.

# Animals and other research organisms

Policy information about studies involving animals; ARRIVE guidelines recommended for reporting animal research, and Sex and Gender in Research

Laboratory animals

Female BALB/c(4–8 weeks old) and Female BALB/c-nu/nu mice (4 and 5 weeks old) were purchased from Japan SLC (Hamamatsu, Japan). Female NOD-SCID (4 and 5 weeks old) and female SCID mice (4 and 5 weeks old) were obtained from Japan Clea (Tokyo, Japan).

Wild animals

The study did not involve wild animals.

Reporting on sex

The information was described in Methods in more details.

Field-collected samples

The study did not involve samples collected from the field.

Ethics oversight

The animal experiments were conducted following the protocols approved by the Institutional Animal Care and Use Committee of Japan Advanced Institute of Science and Technology (No. 07-001). Mice were group-housed in ventilated clear plastic cages under appropriate ambient temperature (~23°C), humidity (~50%), and standard 12 h:12 h light:dark conditions. Experimental group sizes were approved by the regulatory authorities for animal welfare after being defined to balance statistical power, feasibility, and

ethical aspects. Maximal tumour burden permitted is 3000 mm3, and the maximal tumour size/burden was not exceeded in the experiments.

Note that full information on the approval of the study protocol must also be provided in the manuscript.

## Plants

| | |
|---|---|
| Seed stocks | Irrelevant to experiments. |
| Novel plant genotypes | Irrelevant to experiments. |
| Authentication | Irrelevant to experiments. |

## Flow Cytometry

### Plots

Confirm that:

☒ The axis labels state the marker and fluorochrome used (e.g. CD4-FITC).

☒ The axis scales are clearly visible. Include numbers along axes only for bottom left plot of group (a 'group' is an analysis of identical markers).

☒ All plots are contour plots with outliers or pseudocolor plots.

☒ A numerical value for number of cells or percentage (with statistics) is provided.

### Methodology

| | |
|---|---|
| Sample preparation | We have described in Methods in more details. |
| Instrument | MACSQuant® Analyzer 16 (Miltenyi Biotec, Bergisch Gladbach, Germany) |
| Software | MACSQuantify V2.13.3 (Miltenyi Biotec) |
| Cell population abundance | At least 10,000 relevant events were acquired for all FACS analysis. |
| Gating strategy | In general, cells were first gated on FSC/SSC. Singlet cells were gated using FSC-H and FSC-A. Dead cells were then excluded and further surface and intracellular antigen gating was performed on the live cell population. |

☒ Tick this box to confirm that a figure exemplifying the gating strategy is provided in the Supplementary Information.
